# Pharmacological exploitation of the phenothiazine antipsychotics to develop novel antitumor agents–A drug repurposing strategy

**DOI:** 10.1038/srep27540

**Published:** 2016-06-09

**Authors:** Chia-Hsien Wu, Li-Yuan Bai, Ming-Hsui Tsai, Po-Chen Chu, Chang-Fang Chiu, Michael Yuanchien Chen, Shih-Jiuan Chiu, Jo-Hua Chiang, Jing-Ru Weng

**Affiliations:** 1 Institute of Biological Chemistry, Academia Sinica, Taipei 115, Taiwan; 2 College of Medicine, China Medical University, Taichung 404, Taiwan; 3 Division of Hematology and Oncology, Department of Internal Medicine, China Medical University Hospital, Taichung 404, Taiwan; 4 Graduate Institute of Clinical Medical Science, China Medical University, Taichung 404, Taiwan; 5 Institute of Basic Medical Sciences, College of Medicine, National Cheng Kung University, Tainan 701, Taiwan; 6 Cancer Center, China Medical University Hospital, Taichung 404, Taiwan; 7 Department of Oral & Maxillofacial Surgery, China Medical University Hospital, Taichung 404, Taiwan; 8 School of Dentistry, China Medical University, Taichung 404, Taiwan; 9 School of Pharmacy, Taipei Medical University, Taipei 110, Taiwan; 10 Department of Biological Science and Technology, China Medical University, Taichung 404, Taiwan

## Abstract

Phenothiazines (PTZs) have been used for the antipsychotic drugs for centuries. However, some of these PTZs have been reported to exhibit antitumor effects by targeting various signaling pathways *in vitro* and *in vivo*. Thus, this study was aimed at exploiting trifluoperazine, one of PTZs, to develop potent antitumor agents. This effort culminated in A4 [10-(3-(piperazin-1-yl)propyl)-2-(trifluoromethyl)-10*H*-phenothiazine] which exhibited multi-fold higher apoptosis-inducing activity than the parent compound in oral cancer cells. Compared to trifluoperazine, A4 demonstrated similar regulation on the phosphorylation or expression of multiple molecular targets including Akt, p38, and ERK. In addition, A4 induced autophagy, as evidenced by increased expression of the autophagy biomarkers LC3B-II and Atg5, and autophagosomes formation. The antitumor activity of A4 also related to production of reactive oxygen species and adenosine monophosphate-activated protein kinase. Importantly, the antitumor utility of A4 was extended *in vivo* as it, administrated at 10 and 20 mg/kg intraperitoneally, suppressed the growth of Ca922 xenograft tumors. In conclusion, the ability of A4 to target diverse aspects of cancer cell growth suggests its value in oral cancer therapy.

Oral cancer is the sixth leading cause of cancer death worldwide, accounting for over 350,000 deaths per year^1^. The combined use of tobacco, betel nut, and alcohol has been shown to a major risk factor for the development of oral cancer due to their carcinogenic effect in the oral muscosa^2^. Although many therapeutic options are available for the treatment of oral cancer, many patients eventually develop therapeutic resistance. Thus, there is an urgency to develop new chemotherapeutic agents for patients who have developed advanced oral cancer.

Phenothiazines (PTZs) represent a major class of antipsychotic drugs, widely used for the treatment of schizophrenia, and bipolar disorder^3^,^4^. PTZs were originally developed as anti-psychotropic agents due to their inhibitory activity against the dopamine D2 receptor^4^,^5^. Recently, some of these PTZs have been reported to exhibit antitumor effects by targeting various signaling pathways, including those mediated by protein kinase C, calmodulin-dependent enzymes, P-glycoprotein, and protein phosphatase 2A^5^,^6^,^7^. Consequently, recent years have wintnessed the modifications of PTZs to develop different targeted agents, including the triazole derivatives as farnesyltransferase inhibitors^8^ and the *N*-benzoylated derivatives as tubulin inhibitors^9^,^10^,^11^ (Fig. 1A).

In this study, we report the use of trifluoperazine as a lead compound to develop antitumor agents with improved antitumor efficacy and reduced toxicity. This structure optimization effort resulted in a proof-of-concept compound, A4, which exhibited multi-fold higher potency as compared to the parent compound without appreciable cytotoxicity to normal oral human keartinocytes (NHOKs). Mechanistically, A4 retained the activity of trifluoperazine to induce caspase-dependent apoptosis in oral squamous cell carcinoma (OSCC) cells by targeting multiple signaling pathways. Moreover, we obtained evidence that A4 induced autophagic cell death, which might be associated with its ability to activate reactive oxygen species (ROS) production and adenosine monophosphate-activated protein kinase (AMPK). More importantly, A4 was effective *in vivo* in suppressing OSCC xenograft tumor growth, while the trifluoperazine-treated counterparts died within a few days.

## Results

### Structure activity relationship (SAR)

**Figure 1:**
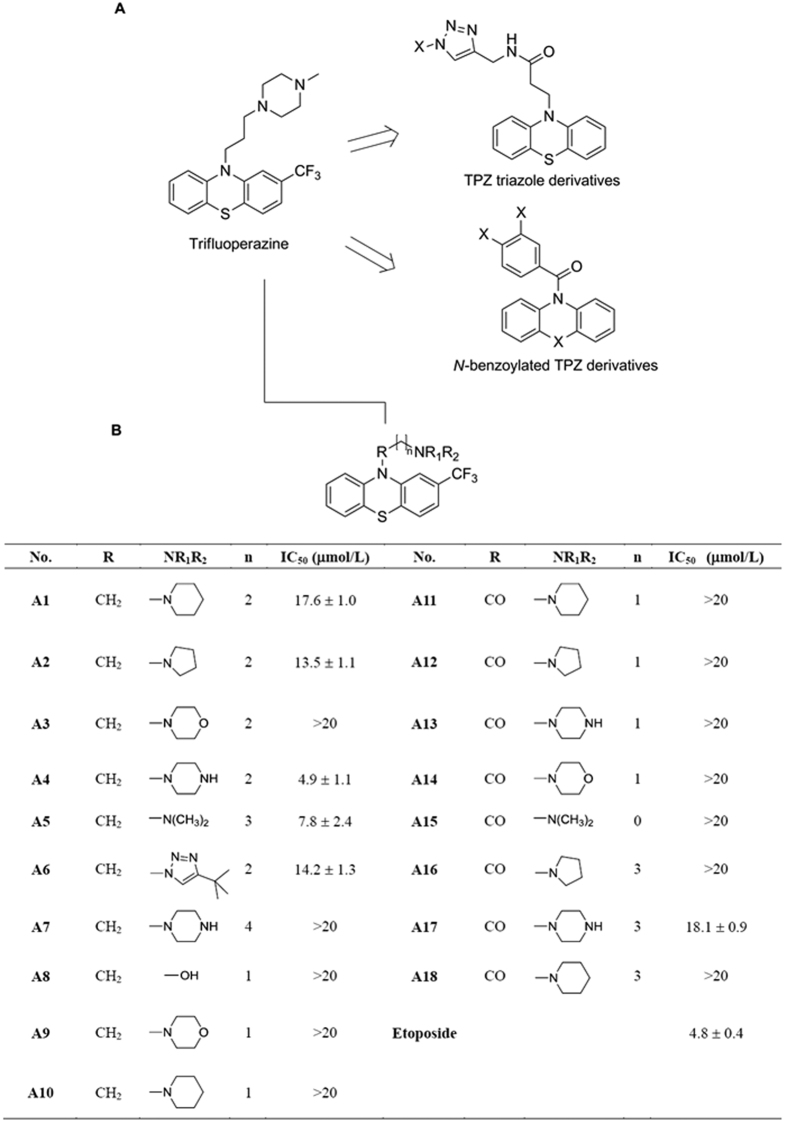
Use of trifluoperazine as scaffolds for developing new anticancer agents. Upper panel, Structure of trifluoperazine, Lower panel, structures and potencies for inducing apoptotic death in Ca922 cells of the trifluoperazine derivatives A1 to A18. Cell viability was assessed by MTT assays with six replicates. The reported IC_50_ values are concentrations at which Ca922 cell death measures 50% relative to DMSO control after 48 h exposure in 5% FBS-containing MEM in 96-well plates.

**Figure 2:**
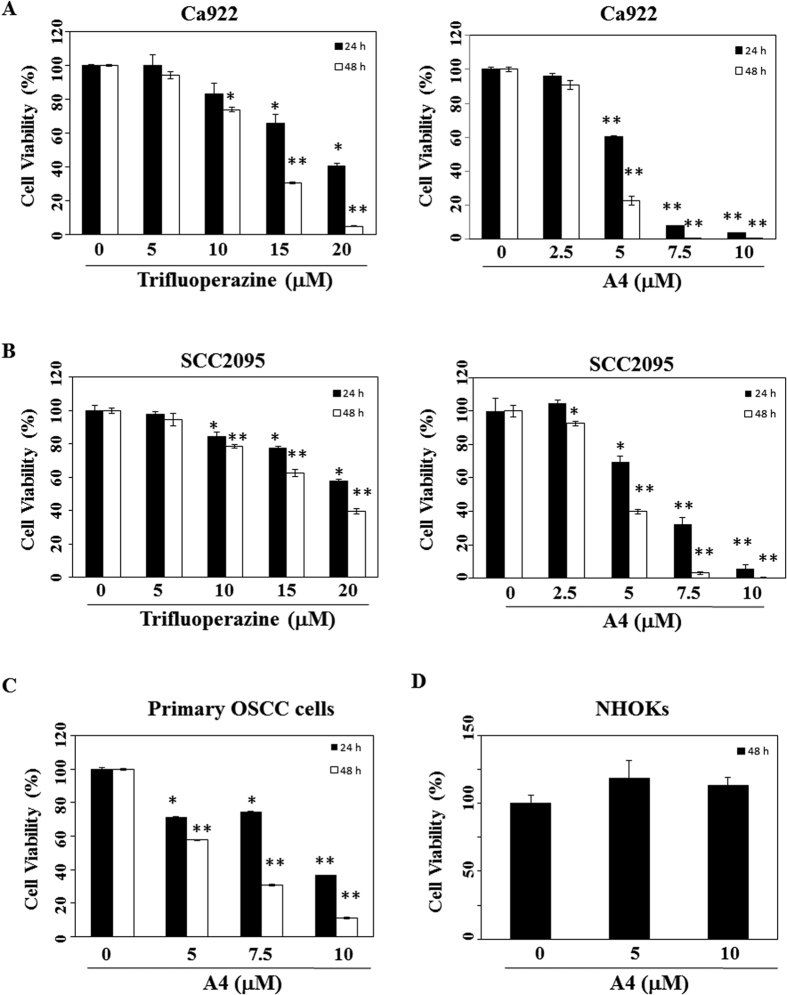
Antiproliferative effects of trifluoperazine and A4 in oral cancer cell lines (Ca922, SCC2095), primary OSCC cells, and NHOKs. (**A**) Ca922, (**B**) SCC2095 (**C**) Primary OSCC cells, and (**D**) NHOKs (5 × 10^3^/200 μL) were treated with DMSO vehicle or trifluoperazine or A4 at the indicated concentrations. Cell viability was assessed by MTT assay as described in the Material section. Points, mean; bars, S.D. (n = 6). *P < 0.05, **P < 0.005 compared to the control group.

In order to improve the antiproliferative effect of trifluoperazine, we synthesized three series of *N*-substituted analogues (Fig. 1B). The antiproliferative activities of these agents (A1–A18) were evaluated in Ca922 oral cancer cells by MTT assays after 48 h of drug treatment. Among these derivatives, A4 exhibited the highest antiproliferative potency with IC_50_ of 4.9 μM (Fig. 1B, etoposide as the positive control), relative to 14 μM for trifluoperazine (Fig. 2A). SAR analysis indicates that replacement of the piperazine ring with a different heterocycle, such as morpholine (i.e., A3) or piperidine (i.e., A10), and/or changes in the length/structure of the linker resulted in substantial loss of antiproliferative potency. Together, these findings suggested that the propyl-piperazine moeity played an integral role in maintaining the antiproliferative activity. This differential antitumor activity was also noted in another OSCC cell line, SCC2095 (IC_50_: A4, 4.5 μM; trifluoperazine, 18 μM, Fig. 2B). We rationalized that this discrepancy was attributable to the *N*-methyl function of trifluoperazine, of which the stereoelectronic effect hindered the ligand interaction with target proteins.

In addition, A4 was effective in suppressing the viability of primary OSCC cells with IC_50_ of 5.6 μM at 48 h (Fig. 2C), while normal humna oral keratinocytes (NHOKs) were insensitive to A4 (Fig. 2D). These findings suggest the discriminative antiproliferative activity of A4 against malignant versus non-malignant oral mucosal cells.

### A4 induces caspase-dependent apoptosis through multiple targets identical to that of trifluoperazine

**Figure 3:**
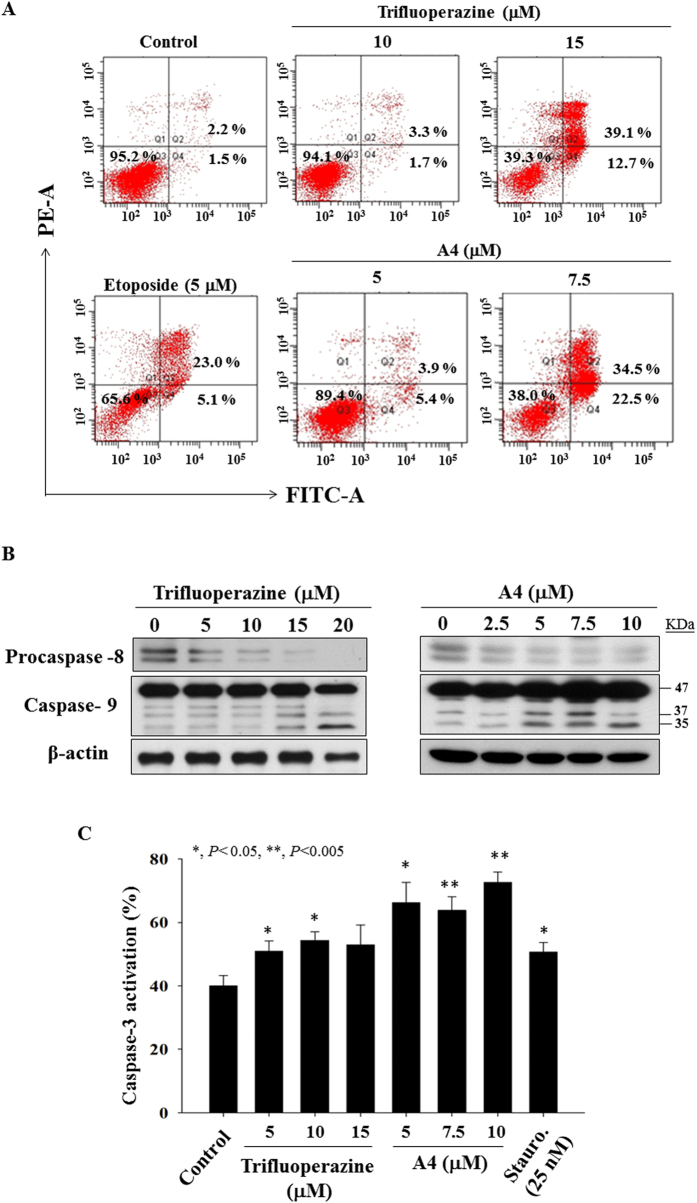
Evidence of apoptosis for trifluoperazine and A4-induced cell death. (**A**) Annexin V-FITC/propidium iodide staining. Ca922 cells were treated with DMSO vehicle or trifluoperazine or A4 at the indicated concentrations in 5% FBS-supplemented MEM medium for 48 h. (**B**) Western blotting of procaspase-8 and caspase-9 after the treatment of trifluoperazine or A4 in Ca922 cells for 48 h. (**C**) Caspase-3 activation of trifluoperazine and A4 in Ca922 cells (n = 4). Staurosporine (Stauro.) as the positive control. *P < 0.05; **P < 0.005 compared to the control group.

We obtained evidence that that A4 retained the ability of trifluoperazine to facilitate apoptotic cell death via caspase activation. For example, annexin V/PI staining showed that treatment of Ca922 cells with either agent led to dose-dependent increases in the apoptotic cell population (Fig. 3A; etoposide as a positive control). This apoptosis induction was confirmed by Western blot and flow cytometric analyses that showed dose-dependent effects of A4 and trifluoperazine on caspase-9 and procapase-8 activation (Fig. 3B) and activated caspase-3 expression (Fig. 3C), respectively.

**Figure 4:**
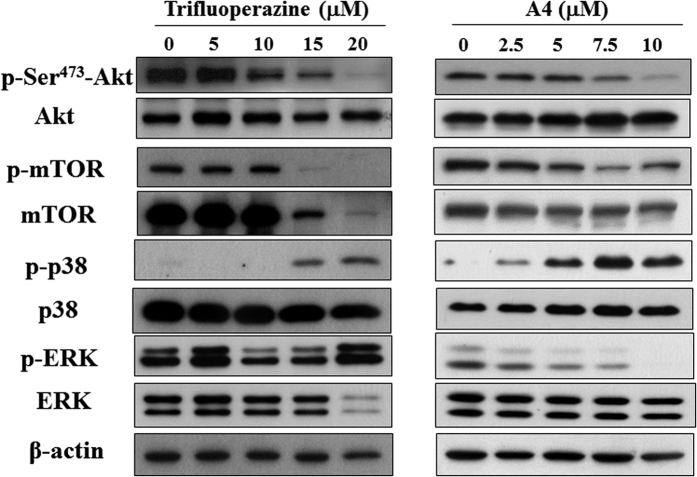
Dose-dependent effects of trifluoperazine and A4 on the phosphorylation/expression of Akt, mTOR, p38, and ERK in Ca922 cells. Cells are treated with trifluoperazine or A4 at the indicated concentrations in 5% FBS-MEM for 48 h and cell lysates were immunoblotted as described in Methods section.

Trifluoperazine has been reported to target multiple signaling pathways to induce apoptosis in cancer cells^12^,^13^. Consequently, we examined the phosporylation status of a series of key signaling kinases, including Akt and its downstream target mTOR, p38, and ERKs. Western blot anaylsis showed that A4 and trifluoperazine dose-dependently decreased the phosphorylation of Akt and mTOR, accompanied by parallel increases in p38 phosphorylation in Ca922 cells (Fig. 4). However, A4 and trifluoperazine diverged on their respective effects on ERK phosphorylation, i.e., only A4 was effective in suppressing p-ERK levels, indicating a subtle difference in their pharmacological properties.

### A4 induces ROS generation and DNA damage

**Figure 5:**
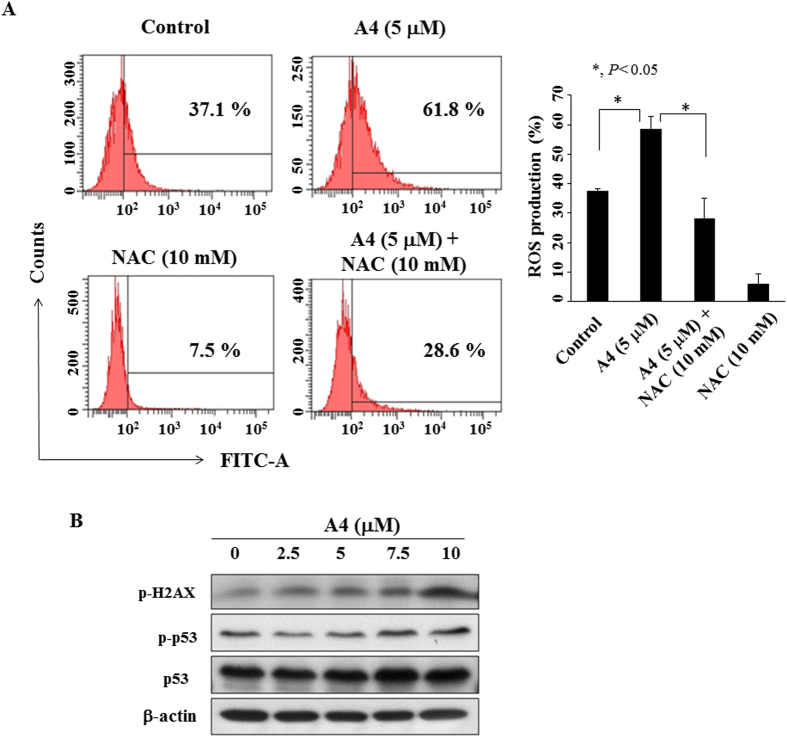
ROS generation of A4 in Ca922 cells. (**A**) Left panel, Flow cytometric analysis of the effect of A4 (5 μM), alone or in combination with the antioxidant *N*-acetylcysteine (NAC) for 3 h on ROS production. Three independent experiments were performed, and the statistical analysis are presented in right panel, Points, mean; bars, S.D. (n = 3). *P < 0.05 compared to the control group. (**B**) Western blotting analysis of the phosphorylation/expression of H2AX and p53 in Ca922 cells.

It has been reported that trifluoperazine inhibited DNA double strand breaks repair in human larynx carcinoma cells and delayed the ability of γ-H2AX resolution in DNA-damaged lung cancer cells^14^,^15^. In light of the mechanistic link between ROS and DNA damage response to many anti-tumor drugs^16^,^17^, we first examined the cellular levels of ROS in A4-treated Ca922 cells (5 μM). As shown in Fig. 5A, 5 μM A4 significantly increased ROS generation in Ca922 cells after 48 h of treatment (37.1% to 61.8%) which, however, could be rescued by *N*-acetylcysteine (NAC), a reported antioxidant^18^. It is noteworthy that this A4-induced ROS generation was accompanied by dose-dependent increases in the phosphorylation of H2AX and p53, hallmarks of DNA damage response^19^,^20^, in Ca922 cells (Fig. 5B).

### A4 induces autophagic cell death

**Figure 6:**
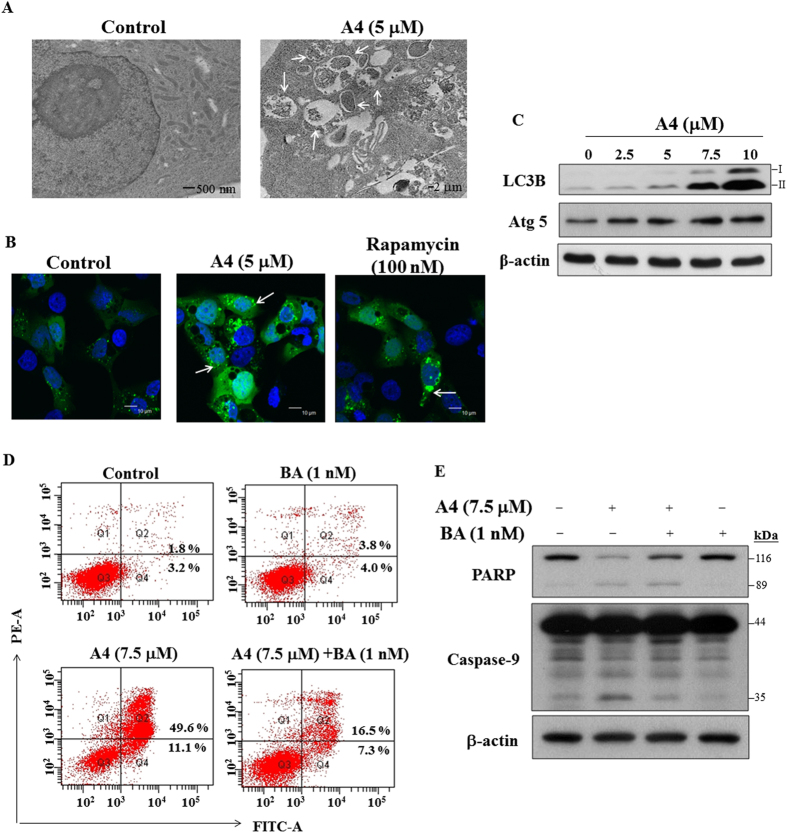
A4 induced autophagy. (**A**) Electron microscopic analysis of autophagosome formation after the treatment of A4 (5 μM) or DMSO in Ca922 cells for 24 h as described in Methods section. Magnification, 12000x. Arrow: autophagosomes. (**B**) Fluorescent confocal microscopic analysis of A4-induced autophagosome formation in Ca922 cells ectopically expressing GFP-LC3. Cells transiently transfected with GFP-LC3 plasmids were treated with DMSO, 5 μM A4, or 100 nM rapamycin for 48 h and then fixed by 3.7% paraldehyde and examined by confocal microscopy. Scale bar: 10 μm. Arrow: autophagosomes. (**C**) Western blotting of LC3B and Atg5 in Ca922 cells treated with A4 for 48 h. (**D**) Ca922 cells were treated with 7.5 μM A4 alone or in combination with 1 nM bafilomycin A1 (BA) for 48 h, and then annexin V-FITC/PI double-staining analysis was performed. (**E**) Western blot analysis of the expression of PARP and caspase-9 after A4 alone or the combination of BA in Ca922 cells.

It has been reported that trifluoperazine induced autophagic degradation without causing cellular damages in glioblastoma cells^21^. Pursuant to this finding, we obtained several lines of evidence to demonstrate the ability of A4 to induce autophagy. First, transmission electron microscopy revealed autophagosome formation in the cytoplasm after exposing Ca922 cells to 5 μM A4 for 24 h (Fig. 6A). Second, Ca922 cells were transiently transfected with GFP-tagged LC3 (GFP-LC3) and exposured 5 μM A4, or 100 nM rapamycin (positive control). Confocal fluorescene imaging demonstrated that the accumulation of LC3-positive puntca in the cytoplasm in a manner similar to that of rapamycin (Fig. 6B). Furthermore, Western blotting showed that A4 dose-dependently increased the expression of LC3B-II and autophagy-related protein (Atg)5 (Fig. 6C), one of the essential molecures which forms pre-autophagosomes^22^,^23^.

To investigate whether this autophagy induction played a protective or cytotoxic role, we examined the effect of the autophagic inhibitor bafilomycin A1 (BA; a vacuolar-type H^+^-ATPase inhibitor) on A4-induced apoptotic cell death. PI/annexin-V analysis demonstrated that co-treatment of Ca922 cells with BA recused A4-induced apoptotic death (Fig. 6D). As shown, Western blotting showed that co-treatment with BA led to a lesser extent of PARP cleavage and caspase-9 activation as compare to A4 alone (Fig. 6E). Together, these data suggested that A4 induces autophagic cell death, which increased the drug effect on apoptosis in Ca922 cells.

### AMPK activation is involved in A4-induced cell death

**Figure 7:**
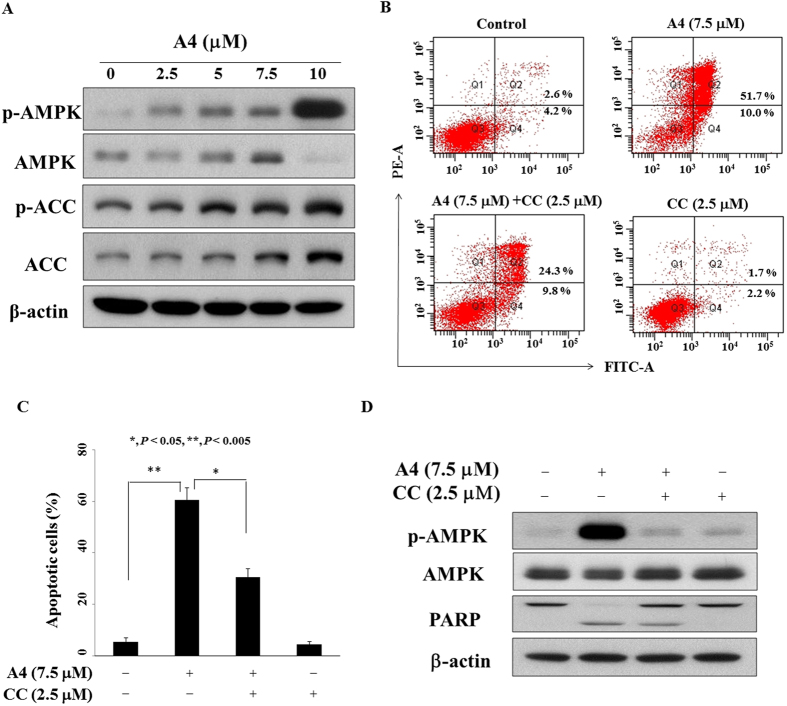
Restoration of antiproliferative activity of A4 by inactivating AMPK. (**A**) The phosphorylation/expression of AMPK and ACC of A4 in Ca922 cells. Cells were treated with A4 in 5% FBS-supplemented MEM medium for 48 h, and cell lysates were immunoblotted as described in Methods. (**B**) Histogram showing 7.5 μM A4 alone or in combination with 2.5 μM compound c (CC) for 48 h, and then annexin V-FITC/PI double-staining analysis was performed. (**C**) The percentage of cells in Q2 and Q4 phases after the treatment was shown. Data are presented as mean ± S.D. *P < 0.05; **P < 0.005. (**D**) Western blotting analysis of the phosphorylation/expression of AMPK and PARP after the combination of CC or A4 alone in Ca922 cells.

In light of the role of AMPK in inducing autophagy through the negative regulation of mTORC1^24^, we further examined the effect of A4 on AMPK activation. Consistent with our premise, Western blotting analysis indicated the ability of A4 to activate AMPK, as manifested by increased phosphorylation levels of AMPK and its downstream target acetyl-CoA carboxylase (ACC) in A4-treated Ca922 cells (Fig. 7A). To clarify the role of AMPK, we examined the effect of the AMPK inhibitor compound c on A4’s antiproliferative activity. Flow cytometric analysis showed that compound c was able to protect Ca922 cells from A4-induced apoptotic cell death (*P* < 0.05; Fig. 7B,C). Furthermore, Western blot analysis showed that co-treatment of compound c abolished the effect of A4 on AMPK activation, which was accompanied by a lesser extent of PARP cleavage relative to A4 alone (Fig. 7C).

### A4 inhibited Ca922 xenograft tumor growth in nude mice

**Figure 8:**
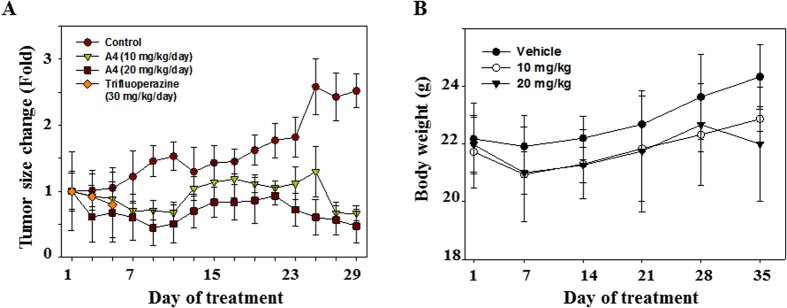
Effects of A4 on Ca922 xenograft tumor size and mice body weight change. (**A**) Mice bearing Ca922 xenografts were treated with normal saline control, A4 (10 mg/kg/day), A4 (20 mg/kg/day), or trifluoperazine (30 mg/kg/day). The tumor size was recorded every 2 days. (**B**) Body weight change of mice.

We examined the *in vivo* antitumor efficacy of A4 relative to trifluoperazine in Ca922 tumor-bearing mice. Daily administration of A4 at 10 or 20 mg/kg by intraperitoneal injection significantly inhibited Ca922 xenograft tumor growth by 74% and 81% (*P* < 0.001), respectively (Fig. 8A). A4 slightly decreased the body weight of tumor-bearing mice during the first week of treatment, however, the differences were not statistically significant and no further decreases were noted in the following weeks (Fig. 8B, *P* = 0.0795). Although mice receiving parent trifluoperazine at 30 mg/kg/day had smaller tumor size compared with mice in control group at initial days (Fig. 8A), all 6 mice in trifluoperazine group had significant body weight loss from 21.2 ± 0.5 mg to 16.5 ± 0.9 mg and died after 5 days of treatment. This suggested a considerable toxicity of trifluoperazine for mice.

## Discussion

As the development of brand-new therapeutic agentes takes an enormous amount of resources, time, and effort, repurposing of existing drugs by exploiting their off-target mechanisms has become a useful strategy for new drug discovery^25^,^26^. In this study, we report the pharmacological exploitation of trifluoperazine, an antipsychotic drug, as a proof-of-concept to develop new antitumor agents for oral cancer therapy. The structurally optimized agent A4 exhibited not only higher *in vitro*/*in vivo* antitumor efficacy, but also lesser toxicity in normal oral human keratinocytes and tumor-bearing mice. Thus, A4 represents a proof-of-concept compound that the phenothiazine class of antipsychotic drugs could be used as starting points for developing novel antitumor agents for clinical development.

The replacement of hydrogen on the piperazine ring endowed A4 with multifold higher apoptosis-inducing potency. Relative to trifluoperazine, A4 displayed similar pattern on modulating multiple molecular targets, including Akt and its downstream effectors mTOR and the MAPK kinases p38 (Fig. 4). It’s noteworthy that these signaling effectors play the important roles in the development of metastsis and invasion in oral cancer cells^27^,^28^,^29^.

In addition, increased oxidative stress including the long-term use of areca nut, dysfunction of antioxidant enzymes, and DNA damage has been implicated in the pathogenesis of oral cancer^30^,^31^,^32^. Interestingly, a recent paper reported that trifluoperazine protected hydrogen peroxide-induced apoptosis in rat pheochromocytoma cells^33^. In contrast, our present study demonstrated that A4 induced ROS generation, which could be rescue by the antioxidant agent NAC. Meanwhile, our finding of A4-induced DNA damage is consistent with the previous report that trifluoperazine impaired DNA repair in lung cancer^34^. Moreover, we found that autophagy is involved in A4-induced cell death, at leat in part, through AMPK activation. Because pharmacological inhibition of AMPK could partially proteced cells from A4-induced apoptosis, we rationalize that this AMPK activation acts in concert with the inhibition of Akt/mTOR and the activation of p38 to facilitate apoptosis in A4-treated oral cancer cells (Fig. 7).

Evidence indicates that autophagy could play a pro-survival or pro-apoptosis role in drug-induced cell death^35^,^36^. It has been reported that trifluoperazine-induced autophagy could protect human dopaminergic cells from wild-type alpha-synuclein-induced toxicity^37^. However, our presente study showed that A4 induced-autophagy played a pro-apoptosis role in oral cancer cells. AMPK, a cellular energy sensor, is one of the molecular targets which modulate autophagy and being the potential target for cancer therapy^38^. Assessment of the *in vivo* efficacy of A4 in tumor-bearing nude mice indicated that A4 was effective in suppressing the growth of established xenograft tumors. More importantly, unlike, its parent compound trifluoperazine, A4 did not incur acute toxicity in tumor-bearing mice.

## Conclusions

Our results show that the trifluoperazine derivative, A4, is a potent antitumor agent in oral cancer cells, which mediates apoptosis, at least in part, through the activation of AMPK, ROS generation, and caspases activation. A4 also induced autophagic cell death, which contributed to its antiproliferative activity. Equally important, A4 exhibited *in vivo* efficacy in suppressing xenograft tumor growth in nude mice without incurring acute toxicity. Taken together, this study provides a proof of concept for the repurposing of PTZ antipsychotic drugs to develop novel therapeutic agents for cancer therapy.

## Methods

### Reagents, Antibodies, and Plasmids

2-(trifluoromethyl)-10*H*-phenothiazine (Sigma-Aldrich, St. Louis, MO, USA) was used as the starting material to synthesize trifluoperazine and trifluoperazine derivatives (Fig. 1A, Figs S3–S16). The identity and purity (>95%) of these synthetic derivatives were identified by proton magnetic resonance spectrometry and HR-EIMS (Figs S5–S38). All agents were dissolved in DMSO, diluted in culture medium, and added to cells at a final DMSO concentration of 0.1%. Antibodies against the following biomarkers were obtained from Cell Signaling Technologies (Danvers, MA, USA): Akt, p-^473^Ser Akt, p-^180/182^Thr/Tyr 38, p38, p-^2448^Ser mTOR, ERK, p-^202/204^Thr/Tyr ERK, LC3B, Atg5, p-^172^Thr AMPK, AMPK, p-^15^Ser p53, p53, p-^139^Ser H2AX, p-^79^Ser ACC, ACC, PARP, procaspase-8, and caspase-9. β-actin was obtained from Sigma-Aldrich. The GFP-LC3 plasmid was purchased from Addgene (Cambridge, MA, USA). The enhanced chemiluminescence system for detection of immunoblotted proteins was from GE Healthcare (Piscataway, NJ, USA). Other chemicals and reagents were obtained from Sigma-Aldrich unless otherwise noted.

### Cell Culture

Ca922 and SCC2095 human oral cancer cells were kindly provided by Professor Susan R. Mallery (The Ohio State University). Ca922 cells were cultured in MEM medium and SCC2095 were cultured in DMEM/F12 medium supplemented with 10% fetal bovine serum (FBS) (Gibco, Grand Island, NY, USA), 5 mg/ml of penicillin and 5 mg/ml of streptomycin. In addition to the above culture condition, 0.4 μg/mL hydrocortisone was added in SCC2095 cells. Primary OSCC cells were isolated from freshly surgical specimens according to a protocol approved by the China Medical University Hospital internal review board. Written informed consent was obtained from all patients in accordance with the Declaration of Helsinki and all experimental procedures were carried out in accordance with the approved protocol. Normal human oral keratinocytes (NHOKs) were kindly provided from Dr. Tzong-Ming Shieh (China Medical University) and were maintained in the keratinocyte serum-free medium (Gibco). All cells were cultured at 37 °C in a humidified incubator containing 5% CO_2_.

### Cell Viability Analysis

The effect of test agents on cell viability was assessed by the 3-(4,5-dimethylthiazol-2-yl)-2,5-diphenyltetrazolium bromide (MTT) assay^39^ in 6 replicates. Briefly, cells (5 × 10^3^) were seeded and incubated in 96-well, flat-bottomed plates in 10% FBS-supplemented MEM or DMEM/F12 or keratinocyte serum-free medium for 24 h, and were exposed to test agents at the indicated concentrations in 5% FBS-supplemented MEM or DMEM/F12 or keratinocyte serum-free medium for different time intervals. Medium was removed and replaced by 200 μL of 0.5 mg/ml MTT in the same medium. After 2 h incubation, the reduced MTT dye was solubilized in 200 μL/well of DMSO. Absorbance was determined with a Synergy HT (Bio-Tek, Winooski, Vermont, USA) at 570 nm.

### Flow Cytometric Analysis

Ca922 cells (5 × 10^4^) were plated and treated with test agents at indicated concentrations in 5% FBS-supplemented MEM medium for 48 h^40^. Cells were harvested, washed twice in ice-cold PBS, fixed in 70% cold ethanol at 4 °C for 4 h, followed by spinning at 1200 rpm for 5 min and re-suspending in ice-cold PBS containing 2% PBS. Cells were stained with annexin V-FITC and PI according to the vendor’s protocols (BD Pharmingen, San Diego, USA) and analyzed by using BD FACSAria flow cytometer (Becton, Dickinson and Co., Franklin Lakes, NY, USA). For caspase-3 activation, drug-treated cells were assessed using a FITC rabbit anti-active caspase-3 kit, according to the vendor’s protocols (BD Pharmingen).

### Western Blotting

Drug-treated cells were collected, washed with ice-cold PBS, and resuspended in lysis buffer [137 mM NaCl, 1 mM CaCl_2_, 20 mM Tris-HCl (pH 8), 0.1% SDS, 100 μM 4-(2-aminoethyl)-benzenesulfonyl fluoride, 0.5% deoxycholate, 10% glycerol, 1% Nonidet P-40, leupeptin at 10 μg/mL, and aprotinin at 10 μg/mL]. Soluble cell lysates were collected after centrifugation at 1500 *g* for 5 min. Equivalent amounts of protein (60–100 μg) from each lysate were resolved in 10% SDS-polyacrylamide gels. Bands were transferred to nitrocellulose membranes and blocked with 5% nonfat milk in PBS containing 0.1% Tween 20 (PBST) and incubated overnight with the corresponding primary antibodies at 4 °C. After washing with PBST three times, the membrane was incubated at room temperature for 1 h with the secondary antibody with PBST and visualized by enhanced chemiluminescence.

### Transient Transfection and Confocal Microscopy

The GFP-LC3 plasmid was transiently transfected into Ca922 cells by using the Fugene HD reagent (Roche, Mannheim, Germany) according to the manufacture’s protocol^41^. Cells (2 × 10^5^/3 mL) were seeded in each well of a six-well plate. After 24 h incubation, cells were treated with A4 (5 μM) or rapamycin (100 nM) as positive control for 15 min, fixed in 2% paraformaldehyde (Merck Millipore, Darmstadt, Germany) for 30 min at room temperature, permeabilized with 0.1% Triton X-100 for 20 min, and washed with PBS. GFP fluorescence was visualized on a Leica TCS SP2 confocal microscope (Leica Biosystems Nussloch GmbH, Heidelberg, Germany).

### Transmission Electron Microscopy

Samples were prepared according to an established procedure^42^. Briefly, Ca922 cells were fixed in a solution containing 0.2 M sodium cacodylate, 2.5% glutaraldehyde, and 2% paraformaldehyde for 1 h. The fixed cells were suspended in a buffered solution containing 1% osmic acid for 1 h, followed by dehydration in a graded ethanol series, wash with acetone, and embedding into EPON epoxy resin. Ultrathin sections (60–80 nm) were prepared on an ultramicrotome and double-stained with uranyl acetate and lead citrate. All sections were examined and photographed with a Hitachi H-600 transmission electron microscope (Hitachi, Tokyo, Japan).

### *In vivo*

Twenty four male nude mice of 5 weeks of age were obtained from the National Laboratory Animal Center (Taipei, Taiwan). Ca922 cells were cultured in MEM supplemented with 10% heat-inactivated FBS. Each mouse was inoculated subcutaneously with 1 × 10^7^ Ca922 cells in 0.1 ml phosphate-buffered saline. Tumor diameter was measured twice weekly using calipers and the tumor volume was calculated using a standard formula: width^2^ × length × 0.52. Body weights of the mice were measured once weekly. When the mean tumor volume reached 60 mm^3^, mice were randomized into four groups (n = 6) that received the following treatments: (a) A4 at 10 mg/kg body weight qd, (b) A4 at 20 mg/kg body weight qd, (c) trifluoperazine at 30 mg/kg body weight qd, and (d) normal saline control. All mice received treatments by intraperitoneal injection (50 μL/mouse) daily till reaching the endpoint. The criteria for endpoint included death, body weight loss more than 30% or tumor size more than 1200 mm^3^. All animal experiments were performed in accordance with the guidelines of the Animal Welfare Act and The Guide for Care and Use of Laboratory Animals from the Council of Agriculture, Executive Yuan. The *in vivo* experiment protocol was approved by the Institutional Animal Care and Use Committee of China Medical University (Taichung, Taiwan, IACUC Approval no.: 103-105-N, period of protocol valid from August 01, 2014 to July 31, 2017).

### Statistical Analysis

All data are presented as mean ± S.D. obtained from three independent experiments. Differences in among group means of tumor volume *in vivo* were analyzed for statistical significance using one-way analysis of variance followed by the Neuman–Keuls test for multiple comparisons. Differences were considered significant *P < 0.05, **P < 0.005. Statistical analyses were performed using SPSS for Windows (SPSS, Chicago, IL, USA).

## Additional Information

**How to cite this article**: Wu, C.-H. *et al.* Pharmacological exploitation of the phenothiazine antipsychotics to develop novel antitumor agents–A drug repurposing strategy. *Sci. Rep.* **6**, 27540; doi: 10.1038/srep27540 (2016).

## Supplementary Material

Supporting Information
