# Supplementary material for: Pharmacological exploitation of the phenothiazine antipsychotics to develop novel antitumor agents–A drug repurposing strategy
Source: Sci Rep. 2016 Jun 9;6:27540. doi: 10.1038/srep27540 (PMC4899727; doi:10.1038/srep27540)
Supplement: Supporting Information [file srep27540-s1.pdf]

Supporting Information

Pharmacological exploitation of the phenothiazine antipsychotics to develop novel antitumor agents – A drug repurposing strategy

Chia-Hsien Wu[1], Li-Yuan Bai[2,3], Ming-Hsui Tsai[4], Po-Chen Chu[1,5], Chang-Fang Chiu[3,6], Michael Yuanchien Chen[7,8], Shih-Jiuan Chiu[9], Jo-Hua Chiang[10], and Jing-Ru Weng[10,*]

[1]Institute of Biological Chemistry, Academia Sinica, Taipei 115, Taiwan, [2]College of Medicine, China Medical University, Taichung 404, Taiwan, [3]Division of Hematology and Oncology, Department of Internal Medicine, China Medical University Hospital, Taichung 404, Taiwan, [4]Graduate Institute of Clinical Medical Science, China Medical University, Taichung 404, Taiwan, [5]Institute of Basic Medical Sciences, College of Medicine, National Cheng Kung University, Tainan 701, Taiwan, [6]Cancer Center, China Medical University Hospital, Taichung 404, Taiwan, [7]Department of Oral & Maxillofacial Surgery, China Medical University Hospital, Taichung 404, Taiwan, [8]School of Dentistry, China Medical University, Taichung 404, Taiwan, [9]School of Pharmacy, Taipei Medical University, Taipei 110, Taiwan, [10]Department of Biological Science and Technology, China Medical University, Taichung 404, Taiwan

Table of Contents

	Page
**General materials and methods for synthesis**	S3
**Procedures for synthesis of compound A1-A18**	S3-S16
**^{1}H NMR spectra of compound A1-A18**	S17-S38

General Materials and Methods for Synthesis

All reagents and solvents were commercial available and used without further purification. Reactions were monitored by thin layer chromatography (TLC) on aluminium backed plates coated with Merck 60 F_{254} silica gel. Flash column chromatography was done using silica gel (Merck Kieselgel 60, No. 9385, 230-400 mesh). ^{1}H NMR spectra were recorded with a Burker AV 300 MHz NMR or a Burker AV 400 MHz or a Burker AV 500 MHz NMR spectrometer. Samples were dissolved in deuterated chloroform ($CDCl_3$) and tetramethylsilane (TMS) was used as a reference. Mass spectra were measured using a JMS-700 from JEOL (Akishima, Japan) or a Premier from Waters Corporation (Massachusetts, USA). Microwave assisted synthesis was carried out in sealed tubes with a CEM Discover SP system (CEM Corporation, Matthews, NC, USA).

Procedures for Synthesis of Compound A1-A18

Scheme1. Synthesis of compound A1-A5, A7[a]

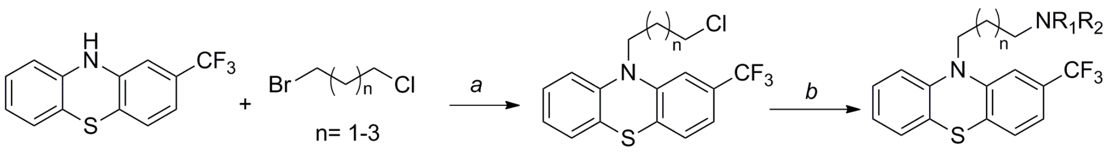

1 + **2-4** → **5-7** → **A1-A5, A7**

Compounds	n	NR_1R_2
**2, 5**	**1**	—
**3, 6**	**2**	—
**4, 7**	**3**	—
**A1**	**1**	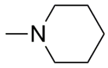
**A2**	**1**	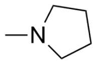
**A3**	**1**	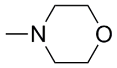
**A4**	**1**	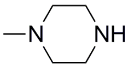
**A5**	**2**	$N(CH_3)_2$
**A7**	**3**	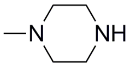

[a]Reagents and conditions: (a) NaH, DMF; (b) acetone, KI, reflux.

General synthetic procedures

Step *a* (Synthesis of 5-7). 2-(trifluoromethyl)-10*H*-phenothiazine (**1**, 2.06g, 10.3 mmol) and sodium hydride (1.21 g, 30.3 mmol) were suspensed in anhydrous DMF (40 ml). After the mixture was stirred for 30 min at room temperature, corresponding Br, Cl-alkane (1-bromo-3-chloropropane or 1-bromo-2-chloropropane or 1-bromo-5-chloropentane, **2-4**, 20 mmol) was added to the reaction and the reaction was stirred for a further 4 hours. 5 ml water was added to the reaction, and the mixture was extracted with ethyl acetate (100 ml x 3). The combined organic layer

was washed with brine, and dried over anhydrous Na_2SO_4. The solvent was removed under vacuum, and the crude product was purified by silica gel column chromatography, eluting with Ethyl acetate/hexanes = 1:10 to give desired compounds (**5-7**) in 83% ~ 93% yield.

Step *b* (Synthesis of A1-A5, A7). The appropriate chloro-trifluoromethylphenothiazine **(5-7**, 0.3 mmol) in acetone (2 ml) was added a catalytic amount of KI and *N,N*-substituted amine (piperidine, pyrrolidine, morpholine, piperazine, or dimethyl amine) (3 mmol). The reaction was stirred at reflux for 12 hr. The solvent was removed under reduced pressure, and the reaction was extracted with ethyl acetate (5 ml x 3). The combined organic layer was washed with brine, dried over anhydrous Na_2SO_4, and concentrated under vacuum. The crude product was purified by flash chromatography (CH_2Cl_2/methanol = 20:1 with 1% $NH_4OH_{(aq)}$) and got the corresponding derivatives (**A1-A5, A7**) in 56% ~ 91% yield.

10-(3-(piperidin-1-yl)propyl)-2-(trifluoromethyl)-10*H*-phenothiazine (A1). Yellow solid, 63% yield. ^{1}H NMR (500 MHz, $CDCl_3$): δ 7.17-7.13 (m, 1H), 7.12-7.10 (m, 2H), 7.09 (dd, *J* = 7.5, 1.3 Hz, 1H), 7.01 (s, 1H), 6.93-6.89 (m, 2H), 3.92 (t, *J* = 6.8 Hz, 2H), 2.46 (t, *J* = 7.2 Hz, 2H), 2.37 (brs, 4H), 1.97 (quintet, *J* = 7.0 Hz, 2H), 1.55 (quintet, *J* = 5.5 Hz, 4H), 1.39 (brs, 2H); HRMS (ESI) *m/z* calcd. for $C_{21}H_{24}N_2F_3S$ $[M+H]^+$: 393.1612, found: 393.1607.

10-(3-(pyrrolidin-1-yl)propyl)-2-(trifluoromethyl)-10*H*-phenothiazine (A2). Yellow solid, 83% yield. ^{1}H NMR (400 MHz, $CDCl_3$): δ 7.29-7.27 (m, 2H), 7.25-7.21 (m, 1H), 7.20 (dd, *J* = 7.7, 1.5 Hz, 1H), 7.07 (s, 1H), 7.02 (td, *J* = 11.3, 1.0 Hz, 1H),

6.97 (dd, *J* = 8.1, 0.8 Hz, 1H), 4.10 (t, *J* = 6.0 Hz, 2H), 3.22-3.15 (m, 6H), 2.50-2.43 (m, 2H), 2.10-2.07 (m, 4H); HRMS (ESI) *m/z* calcd. for $C_{20}H_{22}N_2F_3S$ $[M+H]^+$: 379.1456, found: 379.1452.

4-(3-(2-(trifluoromethyl)-10*H*-phenothiazin-10-yl)propyl)morpholine (A3). Yellow solid, 56% yield. ^{1}H NMR (300 MHz, $CDCl_3$): δ 7.22-7.12 (m, 4H), 7.04 (s, 1H), 6.96 (d, *J* = 14.4 Hz, 1H), 6.94 (d, *J* = 14.9 Hz, 1H), 4.91 (t, *J* = 6.5 Hz, 2H), 3.77 (t, *J* = 4.5 Hz, 4H), 2.67 (t, *J* = 7.2 Hz, 2H), 2.59 (brs, 4 H), 2.10 (quintet, *J* = 6.8 Hz, 2H); HRMS (ESI) *m/z* calcd. for $C_{20}H_{22}N_2OF_3S$ $[M+H]^+$: 395.1405, found: 395.1402.

10-(3-(piperazin-1-yl)propyl)-2-(trifluoromethyl)-10*H*-phenothiazine (A4). Yellow solid, 91% yield. ^{1}H NMR (300 MHz, $CDCl_3$): δ 7.19-7.09 (m, 4H), 7.03 (s, 1H),6.93 (t, *J* = 8.1 Hz, 2H), 3.95 (t, *J* = 6.7 Hz, 2H), 2.82 (t, *J* = 4.7 Hz, 4H), 2.45 (t, *J* = 6.9 Hz, 2H), 2.37 (brs, 4H), 1.92 (quintet, *J* = 6.8 Hz, 2H); HRMS (ESI) *m/z* calcd. for $C_{20}H_{23}N_3F_3S$ $[M+H]^+$: 394.1565 found: 394.1565.

***N,N*-dimethyl-4-(2-(trifluoromethyl)-10*H*-phenothiazin-10-yl)butan-1-amine (A5).** Yellow solid, 71% yield. ^{1}H NMR (300 MHz, $CDCl_3$): δ 7.20-7.11 (m, 4H), 7.05 (s, 1H), 6.97-6.88 (m, 2H), 3.91 (t, *J* = 6.7 Hz, 2H), 2.33 (t, *J* = 7.2 Hz, 2H), 2.22 (s, 6H), 1.84 (quintet, *J* = 7.1 Hz, 2H), 1.64 (quintet, *J* = 7.1 Hz, 2H); HRMS (ESI) *m/z* calcd. for $C_{19}H_{22}N_2F_3S$ $[M+H]^+$: 367.1456, found: 367.1451.

10-(5-(piperazin-1-yl)pentyl)-2-(trifluoromethyl)-10*H*-phenothiazine (A7). Yellow solid, 77% yield. ^{1}H NMR (500 MHz, $CDCl_3$): δ 7.20-7.15 (m, 2H), 7.15-7.11 (m, 2H), 7.00 (s, 1H), 6.96-6.93 (m, 1H), 6.86 (d, *J* = 8.2 Hz, 1H), 3.87 (t, *J* = 6.9 Hz, 2H), 2.89 (t, *J* = 4.8 Hz, 4H), 2.39 (brs, 4H), 2.30 (t, *J* = 7.4 Hz, 2H), 1.81 (quintet, *J* = 7.2 Hz, 2H), 1.57-1.42 (m, 4H); HRMS (ESI) *m/z* calcd. for $C_{22}H_{27}N_3F_3S$ $[M+H]^+$: 422.1878, found: 422.1873.

Scheme 2. Synthesis of compound A9-A14[a]

General synthetic procedures

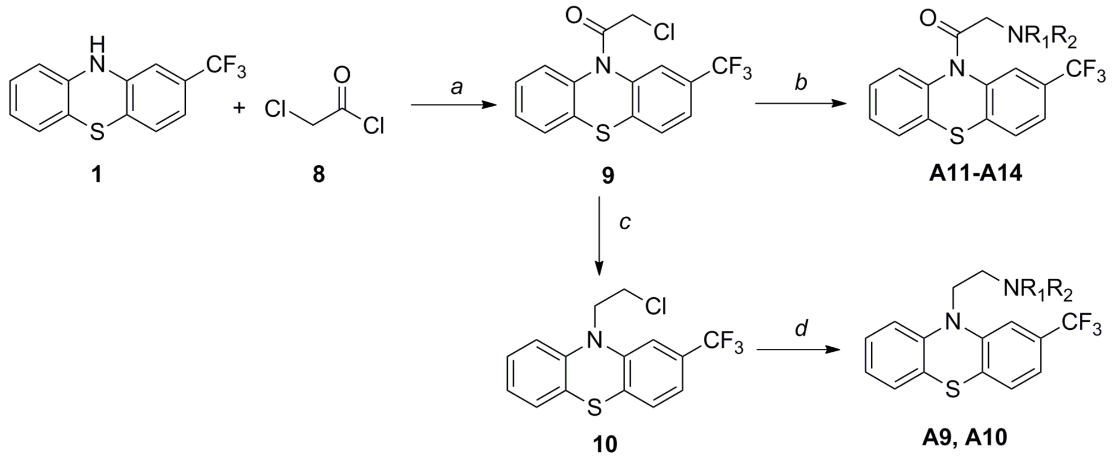

Compounds	NR_1R_2
**A9, A14**	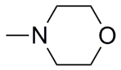
**A10, A11**	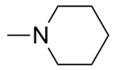
**A12**	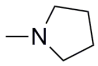
**A13**	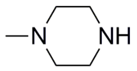

[a]Reagents and conditions: (a) PhCl, microwave irradiation; (b) PhCl, 100 °C; (c) $NaBH_4$, $BF_3{\cdot}Et_2O$ (d) acetone, KI, reflux.

Step *a* (Synthesis of 9 [2-chloro-1-(2-(trifluoromethyl)-10*H*-phenothiazin-10-yl)ethanone]**).** 2-(trifluoromethyl)-10*H*-phenothiazine (**1**, 500 mg, 1.87 mmol) in chlorobenzene (5 ml) was added 2-chloroacetyl chloride (**8**, 528 mg, 4.67 mmol) dropwise. The mixture was stirred for 10 min and heated to 120 °C under microwave irradiation for 30 min. The reaction was extracted with ethyl acetate (10 ml x 3), washed with brine, dried over anhydrous Na_2SO_4, and concentrated under reduced pressure. The crude was purified by silica gel column chromatography (ethyl acetate/ hexanes = 1:20) and pale yellow solid was obtained (**9**, 629 mg; yield: 98.1%). ^{1}H

NMR (300 MHz, $CDCl_3$): δ 7.90 (s, 1H), 7.57-7.47 (m, 4H), 7.38 (td, J = 11.3, 1.5 Hz, 1H), 7.34-7.29 (m, 1H), 4.24, 4.13 (ABq, J_{AB} = 12.6 Hz, 2H).

Step *b* (Synthesis of A11-14). A mixture of **9** (100 mg, 0.37 mmol), chlorobenzene, and the corresponding amine (piperidine, pyrrolidine, piperazine, or mopholine) (2 mmol) was stirred at 100 °C for 8 h. The mixture was cooled to room temperature, and then extracted with ethyl acetate (10 ml x 3), washed with brine, dried over anhydrous Na_2SO_4, and concentrated under reduced pressure. The crude was purified by silica gel column chromatography (CH_2Cl_2/methanol = 40:1 with 1%$NH_4OH_{(aq)}$) and desired product (**A11-A14**) was obtained (yield: 56 ~ 80%).

Step *c* (Synthesis of 10). A mixture of **9** (500 mg, 1.46 mmol) in anhydrous THF (8 ml) was added boron trifluoride diethyl etherate (0.27 ml, 2.19 mmol) dropwise at 0 °C. $NaBH_4$ (66 ml, 1.75 mmol) was added to the mixture and stirred for 1 h at 0 °C. The reaction was quenched by the addition of 5 ml of saturated aqueous $NaHCO_3$. The mixture was extracted with ethyl acetate (10 ml x 3), washed with brine, dried over anhydrous Na_2SO_4, and concentrated under reduced pressure. The crude product was purified by silica gel column chromatography, eluting with ethyl acetate/hexanes = 1:100 to afford the product **10** [10-(2)-chloroethyl)-2-(trifluoromethyl)-10*H*-phenothiazine] (340 mg, yield: 70.9%) as a white solid.^{1}H NMR (300 MHz, $CDCl_3$): δ 7.24-7.16 (m, 3H), 7.16-7.13 (m, 1H), 7.03 (brs, 1H),

7.02-6.96 (m, 1H), 6.89 (d, *J* = 8.1 Hz, 1H), 4.25 (t, *J* = 7.1 Hz, 2H), 3.78 (t, *J* = 7.1 Hz, 2H).

Step *d* (Synthesis of A9-A10). A mixture of **10** (100 mg, 0.30 mmol), DMF (2 ml), KI and *N,N*-substituted amine (morpholine or piperazine) (1.5 mmol) was stirred in a flask for 8 hat 100 ℃. The reaction was quenched by adding NH_4Cl solution (5 ml), extracted with ethyl acetate (10 ml x 3), washed with brine, dried over anhydrous Na_2SO_4, and concentrated under reduced pressure. The crude was purified by silica gel column chromatography, eluting with CH_2Cl_2/methanol = 40:1 with 1%$NH_4OH_{(aq)}$ to give the desired products (**A9-A10**). Yield: 70 ~ 86%.

4-(2-(2-(trifluoromethyl)-10*H*-phenothiazin-10-yl)ethyl)morpholine (A9). White solid, 70% yield. ^1H NMR (300 MHz, $CDCl_3$): δ 7.25 (s, 1H), 7.19-7.09 (m, 4H), 6.93 (td, *J* = 11.2, 1.0 Hz, 1H), 6.89 (d, *J* = 8.1 Hz, 1H), 4.03 (t, *J* = 6.4 Hz, 2H), 3.73 (t, *J* = 4.5 Hz, 4H), 2.76 (t, *J* = 6.4 Hz, 2H), 2.54 (t, *J* = 4.4 Hz, 4H); HRMS (ESI) *m/z* calcd. for $C_{19}H_{20}N_2OF_3S$ $[M+H]^+$: 381.1248, found: 381.1247.

10-(2-(piperidin-1-yl)ethyl)-2-(trifluoromethyl)-10*H*-phenothiazine (A10). Yellow solid, 86% yield. ^1H NMR (300 MHz, $CDCl_3$): δ 7.28 (s, 1H), 7.18-7.08 (m, 4H), 6.93 (t, *J* = 8.3 Hz, 2H), 4.01 (t, *J* = 6.7 Hz, 2H), 2.71 (t, *J* = 6.7 Hz, 2H), 2.48 (brs, 4H), 1.63-1.58 (m, 4H), 1.46-1.45 (m, 2H); HRMS (ESI) *m/z* calcd. for $C_{20}H_{22}N_2F_3S$ $[M+H]^+$: 379.1456, found: 379.1447.

2-(piperidin-1-yl)-1-(2-(trifluoromethyl)-10*H*-phenothiazin-10-yl)ethan-1-one (A11). Yellow solid, 61% yield. ^1H NMR (300 MHz, $CDCl_3$): δ 7.91 (s, 1H), 7.56-7.41 (m, 4H), 7.33 (td, *J* = 7.6, 1.3 Hz, 1H), 7.27-7.21 (m, 1H), 3.25 (q, *J* = 12.6 Hz, 2H), 2.32 (t, *J* = 5.0 Hz, 4H), 1.40-1.25 (m, 6H); HRMS (ESI) *m/z* calcd. for $C_{20}H_{20}N_2OF_3S$ $[M+H]^+$: 393.1248, found: 393.1241.

2-(pyrrolidin-1-yl)-1-(2-(trifluoromethyl)-10*H*-phenothiazin-10-yl)ethan-1-one (A12). Yellow solid, 56% yield. ^{1}H NMR (300 MHz, $CDCl_3$): δ 7.97 (s, 1H), 7.57 (dd, *J* = 7.9, 1.0 Hz, 1H), 7.51 (d, *J* = 8.1Hz, 1H), 7.43 (td, *J* = 11.4, 1.1 Hz, 2H), 7.33 (td, *J* = 11.4, 1.5 Hz, 1H), 7.27-7.22 (m, 1H), 3.43 (brs, 1H), 3.34 (brs, 1H), 2.56 (brs, 4H), 1.73 (quintet, *J* = 3.2 Hz, 4H); HRMS (ESI) *m/z* calcd. for $C_{19}H_{18}N_2OF_3S$ $[M+H]^+$: 379.1092, found: 379.1086.

2-(piperazin-1-yl)-1-(2-(trifluoromethyl)-10*H*-phenothiazin-10-yl)ethan-1-one (A13). Yellow solid, 80% yield.^{1}H NMR (300 MHz, $CDCl_3$): δ 7.83 (s, 1H), 7.58-7.52 (m, 1H), 7.48-7.46 (m, 3H), 7.36-7.25 (m, 2H), 3.38 (d, *J* = 4.1 Hz, 1H), 3.35 (d, *J* = 3.6 Hz, 1H), 3.02 (brs, 4H), 2.68 (brs, 4H); HRMS (ESI) *m/z* calcd. for $C_{19}H_{19}N_3OF_3S$ $[M+H]^+$: 394.1201, found: 394.1198.

2-morpholino-1-(2-(trifluoromethyl)-10*H*-phenothiazin-10-yl)ethan-1-one (A14). White solid, 77% yield. ^{1}H NMR (300 MHz, $CDCl_3$): δ 7.92 (s, 1H), 7.55 (d, *J* = 8.1 Hz, 2H), 7.46 (t, *J* = 7.9 Hz, 2H), 7.34 (td, *J* = 11.2, 1.4 Hz, 1H), 7.29-7.24 (m, 1H), 3.56 (t, *J* = 4.5 Hz, 4H), 3.32, 3.25 (ABq, J_{AB} = 13.8 Hz, 2H), 2.20 (t, *J* = 4.5 Hz, 4H); HRMS (ESI) *m/z* calcd. for $C_{19}H_{18}N_2O_2F_3S$ $[M+H]^+$: 395.1041, found: 395.1043.

Scheme 3. Synthesis of compound A16-A18[a]

General synthetic procedures

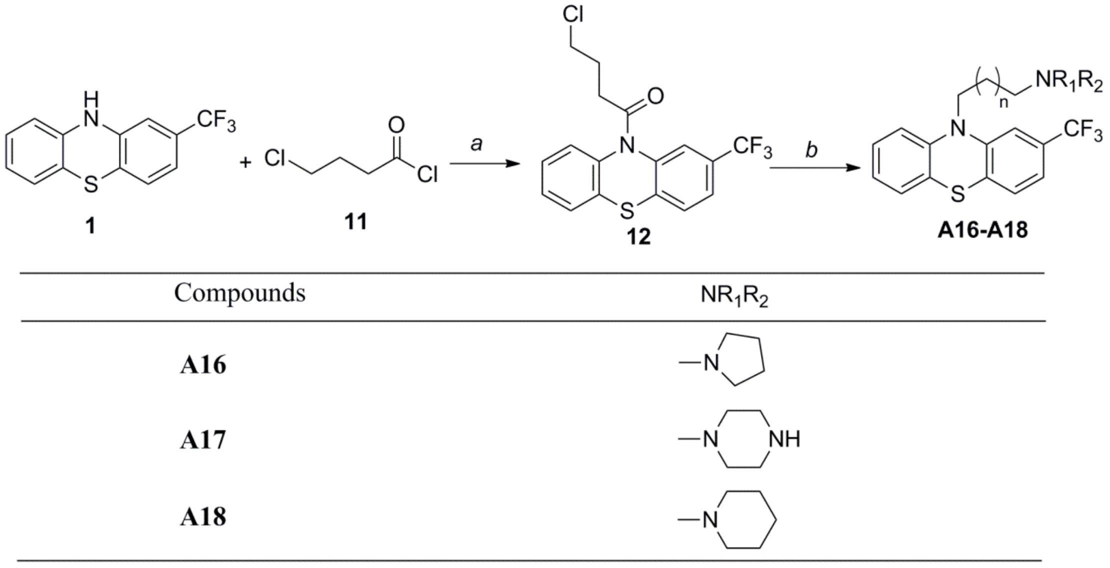

[a]Reagents and conditions: (a) PhCl, microwave irradiation; (b) PhCl, 100 °C.

Step *a* (Synthesis of 12 [4-chloro-1-(2-(trifluoromethyl)-10*H*-phenothiazin-10-yl)butan-1-one]**).** A mixture of **1** (1000 mg, 3.74 mmol), chlorobenzene (5 ml), and 4-chlorobutanoyl chloride (**11**, 1.05g, 9.35 mmol) was heated to 120 °C under microwave irradiation for 1h. The mixture was extracted with ethyl acetate (20 ml x 3), washed with brine, dried over anhydrous Na_2SO_4, and concentrated under reduced pressure. The crude was purified by silica gel column chromatography (ethyl acetate/ hexanes = 1:4) to get the product **12** (1.1 g, yield: 80.2%) as a white solid. ^{1}H NMR (300 MHz, $CDCl_3$): δ 7.82 (s, 1H), 7.56-7.45 (m, 4H), 7.37 (td, *J* = 11.4, 1.4 Hz, 1H), 7.30 (dd, *J* = 7.6, 1.3 Hz, 1H), 3.57 (q, *J* = 5.9 Hz, 2H), 2.75-2.53 (m, 2H), 2.14-2.05 (m, 2H).

Step *b* (Synthesis of A16-A18). A mixture of **12** (100 mg, 0.27 mmol), chlorobenzene (2 ml), the corresponding amine (pyrrolidine, piperazine, or piperidine) (2 mmol), was stirred at 100 °C for 8 h. The mixture was cooled to room temperature, and extracted with ethyl acetate (10 ml x 3), washed with brine, dried over anhydrous Na_2SO_4, and concentrated under reduced pressure. The crude was purified by silica gel column chromatography (CH_2Cl_2/methanol = 20:1 with 1%$NH_4OH_{(aq)}$) to give the product (**A16-A18**). Yield: 74%~85%.

4-(pyrrolidin-1-yl)-1-(2-(trifluoromethyl)-10*H*-phenothiazin-10-yl)butan-1-

one (A16). Yellow solid, 81% yield. ^{1}H NMR (300 MHz, $CDCl_3$): δ 7.77 (s, 1H), 7.49-7.38 (m, 4H), 7.29 (td, *J* = 11.4, 1.3 Hz, 1H), 7.24-7.10 (m, 1H), 2.51-2.39 (m, 8H), 1.81 (quintet, *J* = 7.1 Hz, 2H), 1.70 (brs, 4H); HRMS (APCI) *m/z* calcd. for $C_{21}H_{22}N_2OF_3S$ $[M+H]^+$: 407.1405, found 407.1404.

4-(piperazin-1-yl)-1-(2-(trifluoromethyl)-10*H*-phenothiazin-10-yl)butan-1-one (A17). Yellow solid, 74% yield. ^{1}H NMR (300 MHz, $CDCl_3$): δ 7.83 (s, 1H), 7.55-7.44 (m, 4H), 7.35 (td, *J* = 11.2, 1.2 Hz, 1H), 7.30-7.25 (m, 1H), 2.83 (t, *J* = 9.4 Hz, 4H), 2.53-2.50 (m, 4H), 2.34 (brs, 3H), 2.27 (t, *J* = 7.0 Hz, 2H), 1.82 (quintet, *J* = 6.9 Hz, 2H); HRMS (ESI) *m/z* calcd. for $C_{21}H_{23}N_3OF_3S$ $[M+H]^+$: 422.1514, found: 422.1513.

4-(piperidin-1-yl)-1-(2-(trifluoromethyl)-10*H*-phenothiazin-10-yl)butan-1-one (A18). Yellow solid, 85% yield. ^{1}H NMR (300 MHz, $CDCl_3$): δ 7.83 (s, 1H), 7.56-7.44 (m, 4H), 7.37 (td, *J* = 7.5, 1.0 Hz, 1H), 7.30-7.26 (m, 1H), 2.49 (brs, 8H), 1.93 (quintet, *J* = 6.9 Hz, 2H), 1.66 (brs, 4H), 1.46 (brs, 2H); HRMS (ESI) *m/z* calcd. for $C_{22}H_{24}N_2OF_3S$ $[M+H]^+$: 421.1561, found: 421.1559.

Scheme 4. Synthesis of compound A6[a]

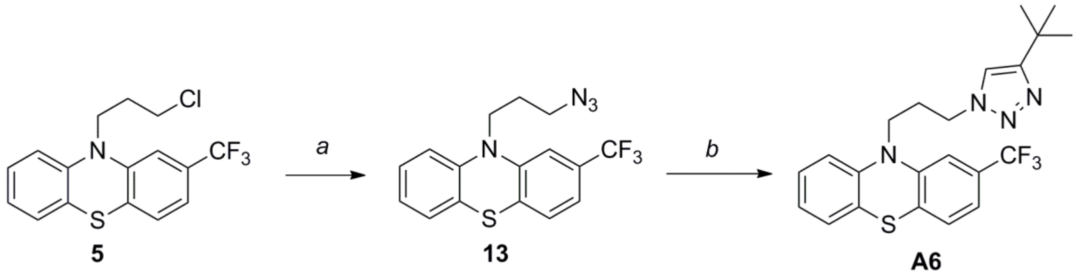

[a]Reagents and conditions: (a) NaN_3, DMF, 90 °C; (b) $CuSO_4$, sodium ascorbate, 3,3-dimethyl-1-butyne, *t*-BuOH/ H_2O, rt.

Step *a* (Synthesis of 13). To a solution of10-(3-chloropropyl)-2-(trifluoromethyl)-10*H*-phenothiazine (**5**) (1.5 g, 4.35 mmol) dissolved in anhydrous DMF (20 ml) was added sodium azide (564 mg, 8.7 mmol) and KI (722 mg, 4.35 mmol). The mixture was stirred at 90℃ for 12 h followed by adding water to quench. The mixture was extracted with ethyl acetate (30 ml x 3), washed with brine, dried

over anhydrous Na_2SO_4, and concentrated under reduced pressure. The crude was purified by silica gel column chromatography, eluting with ethyl acetate/hexanes = 1:20, and give product **13** [10-(3-azidopropyl)-2-(trifluoromethyl)-10*H*-phenothiazine] (1.5 g, yield: 98.5%) as a yellow oil. ^{1}H NMR (500 MHz, $CDCl_3$): δ 7.24-7.23 (m, 1H), 7.22-7.15 (m, 3H), 7.05 (s, 1H), 6.97 (td, *J* = 11.7, 0.8 Hz, 1H), 6.91 (d, *J* = 7.9 Hz, 1H), 4.02 (t, *J* = 6.5 Hz, 2H), 3.45 (t, *J* = 6.3 Hz, 2H), 2.03 (quintet, *J* = 6.4 Hz, 2H).

Step *b* (Synthesis of A6). A mixture of **13** (150 mg, 0.53 mmol), $CuSO_4$ (27.5 mg, 0.11 mmol), sodium ascorbate (43.6 mg, 0.22 mmol), and 3,3-dimethyl-1-butyne (87 mg, 1.06 mmol) was dissolved in *t*-BuOH/H_2O = 1:1 (5 ml), stirred for 14 h at room temperature. The reaction was extracted with ethyl acetate (10 ml x 3), washed with brine, dried over anhydrous Na_2SO_4, and concentrated under reduced pressure. The crude product was purified by silica gel column chromatography, eluting with ethyl acetate/hexanes = 1:8 to give 160 mg of **A6**.

10-(3-(4-(tert-butyl)-1H-1,2,3-triazol-1-yl)propyl)-2-(trifluoromethyl)-10*H*-phenothiazine (A6). White solid, 87% yield. ^{1}H NMR (300 MHz, $CDCl_3$): δ 7.29 (t, *J* = 7.3 Hz, 1H), 7.23-7.16 (m, 3H), 7.04-6.97 (m, 3H), 6.83 (d, *J* = 8.0 Hz, 1H), 4.37 (t, *J* = 6.1 Hz, 2H), 3.76 (t, *J* = 5.91 Hz, 2H), 2.42 (quintet, *J* = 6.0 Hz, 2H), 1.12 (s, 9H); HRMS (ESI) *m/z* calcd. for $C_{22}H_{23}N_4F_3NaS$ $[M+Na]^+$: 455.1493, found: 455.1486.

Scheme 5. Synthesis of compound A8[a]

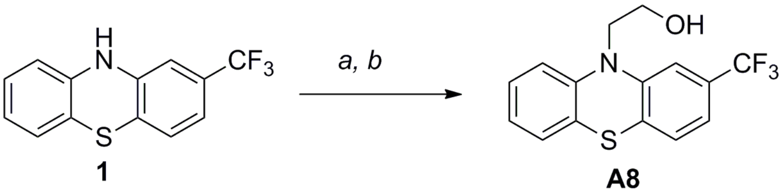

[a]Reagents and conditions: (a) *n*-BuLi, THF, -78 °C; (b) ethlene oxide

Synthetic procedures of A8

2-(trifluoromethyl)-10*H*-phenothiazine (**1**) (480 mg, 1.80 mmol) in anhydrous THF (15 ml) was added *n*-BuLi (2.5 M, 0.72 ml) dropwise at -78 ℃, and the mixture was stirred for 1 h. Ethylene oxide (2.5M~3.3M, 0.6 ml) was added to the reaction and allowed to warm up to room temperature, followed by stirring for further 4 h. The reaction was quenched by addition of water (1 ml), extracted with ethyl acetate (20 ml x 3), washed with brine, dried over anhydrous Na_2SO_4, and concentrated under reduced pressure. The crude was purified by silica gel column chromatography, eluting with ethyl acetate/hexanes = 1:10. 280 mg of **A8** was obtained.

2-(2-(trifluoromethyl)-10*H*-phenothiazin-10-yl)ethan-1-ol (A8). White solid, 50% yield. ^{1}H NMR (300 MHz, $CDCl_3$): δ 7.28-7.26 (m, 1H), 7.24-7.17 (m, 3H), 7.10 (s, 1H), 6.99 (td, *J* = 11.3, 1.1 Hz, 1H), 6.94 (d, *J* = 8.1 Hz, 1H), 4.15 (t, *J* = 5.3 Hz, 2H), 3.93 (q, *J* = 5.4 Hz, 2H); HRMS (ESI) *m/z* calcd. for $C_{15}H_{11}NOF_3S$ $[M-H]^-$: 310.0513, found: 310.0504.

Scheme 5. Synthesis of compound A15

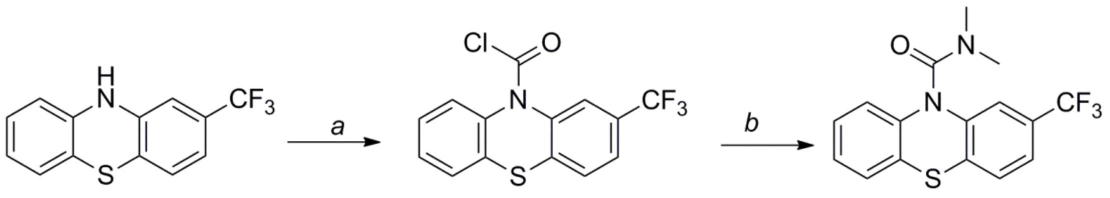

1 14 A15

[a]Reagents and conditions: (a) phosgen, THF/toluene; (b) dimethyl amine

Synthetic procedures of A15

Step *a* (Synthesis of 14). 2-(trifluoromethyl)-10*H*-phenothiazine (**1**) (1.07 g, 4 mmol) and pyridine (474 mg, 6 mmol) in toluene/THF = 1:1 (30 ml) was added phosgen (15% in toluene, 4.4 ml) dropwise, followed by stirring for another 12 h. The reaction was quenched by adding methanol (1 ml) and concentrated under vacuum. The resin was extracted with ethyl acetate (20 ml x 3), washed with brine, dried over anhydrous Na_2SO_4, and concentrated under reduced pressure. The crude was purified by silica gel column chromatography, eluting with ethyl acetate/hexanes = 1:20 to afford 1.1 g of **14** [2-(trifluoromethyl)-10*H*-phenothiazine-10-carbonyl chloride] as a yellow solid (83.5% yield). ^{1}H NMR (300 MHz, $CDCl_3$): δ 7.88 (s, 1H), 7.63-7.51 (m, 3H), 7.48-7.37 (m, 2H), 7.33-7.25 (m, 1H).

Step *b* (Synthesis of A15). To a solution of **14** (100 mg, 0.30 mml) dissolved in MeOH (2 ml) was added dimethyl amine in MeOH (0.5 ml). After stirring for 6 h at room temperature, the solvent was evaporated in vacuo. The mixture was extracted with ethyl acetate (10 ml x 3), washed with brine, dried over anhydrous Na_2SO_4, and concentrated under reduced pressure. The crude was purified by silica gel column

chromatography, eluting with ethyl acetate/hexanes = 1:10. 90 mg of **A15** was obtained.

***N,N*-dimethyl-2-(trifluoromethyl)-10*H*-phenothiazine-10-carboxamide (A15).** Yellow solid, 88% yield. ^{1}H NMR (300 MHz, $CDCl_3$): δ 8.10 (s, 1H), 7.36-7.28 (m, 4H), 7.22 (d, *J* = 8.0 Hz, 1H), 7.10 (t, *J* = 7.5 Hz, 1H), 2.88 (s, 6H); HRMS (FAB) *m/z* calcd. for $C_{16}H_{13}N_2OF_3S$ $[M]^+$: 338.0701, found: 338.0707.

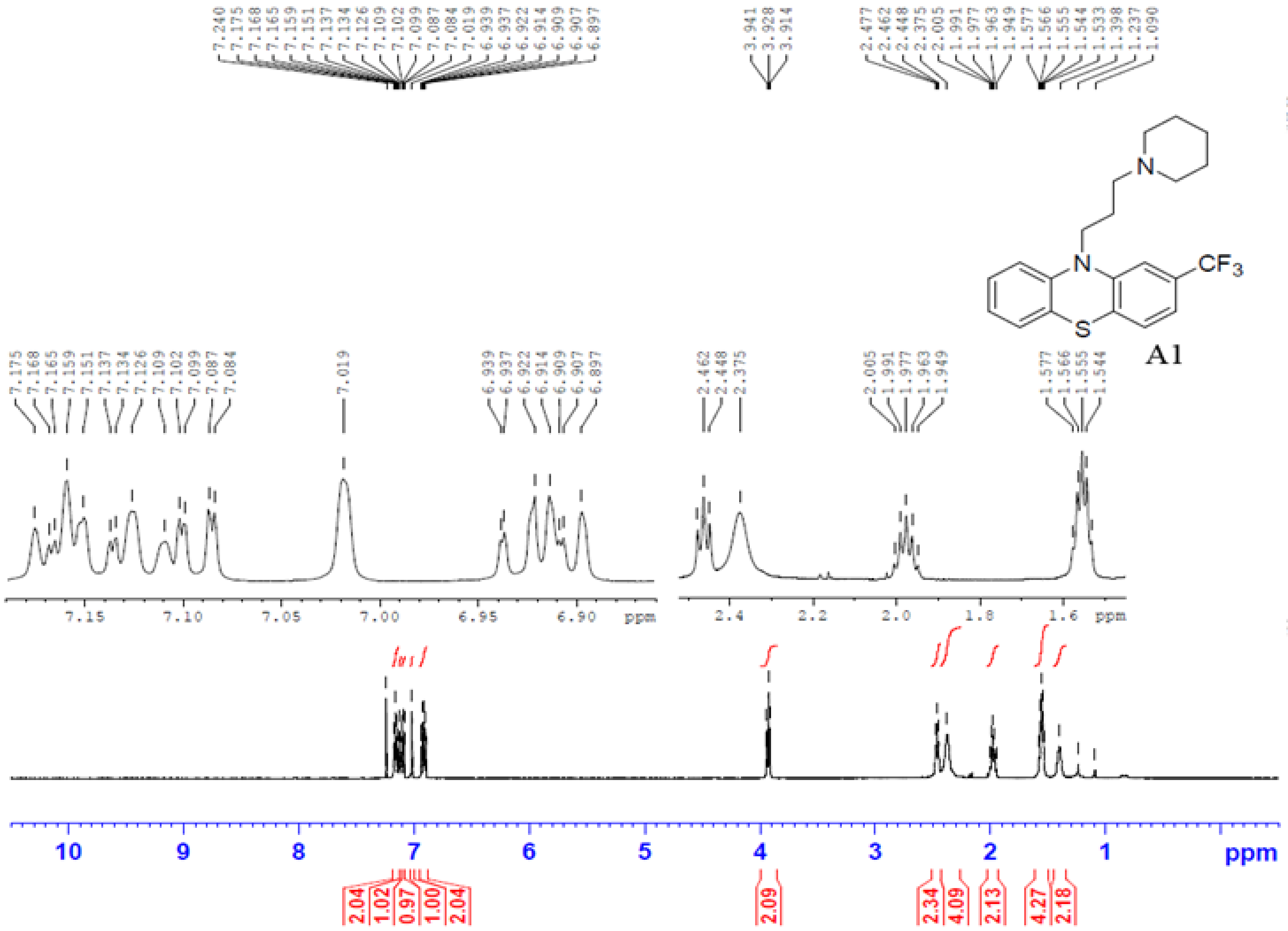

A1

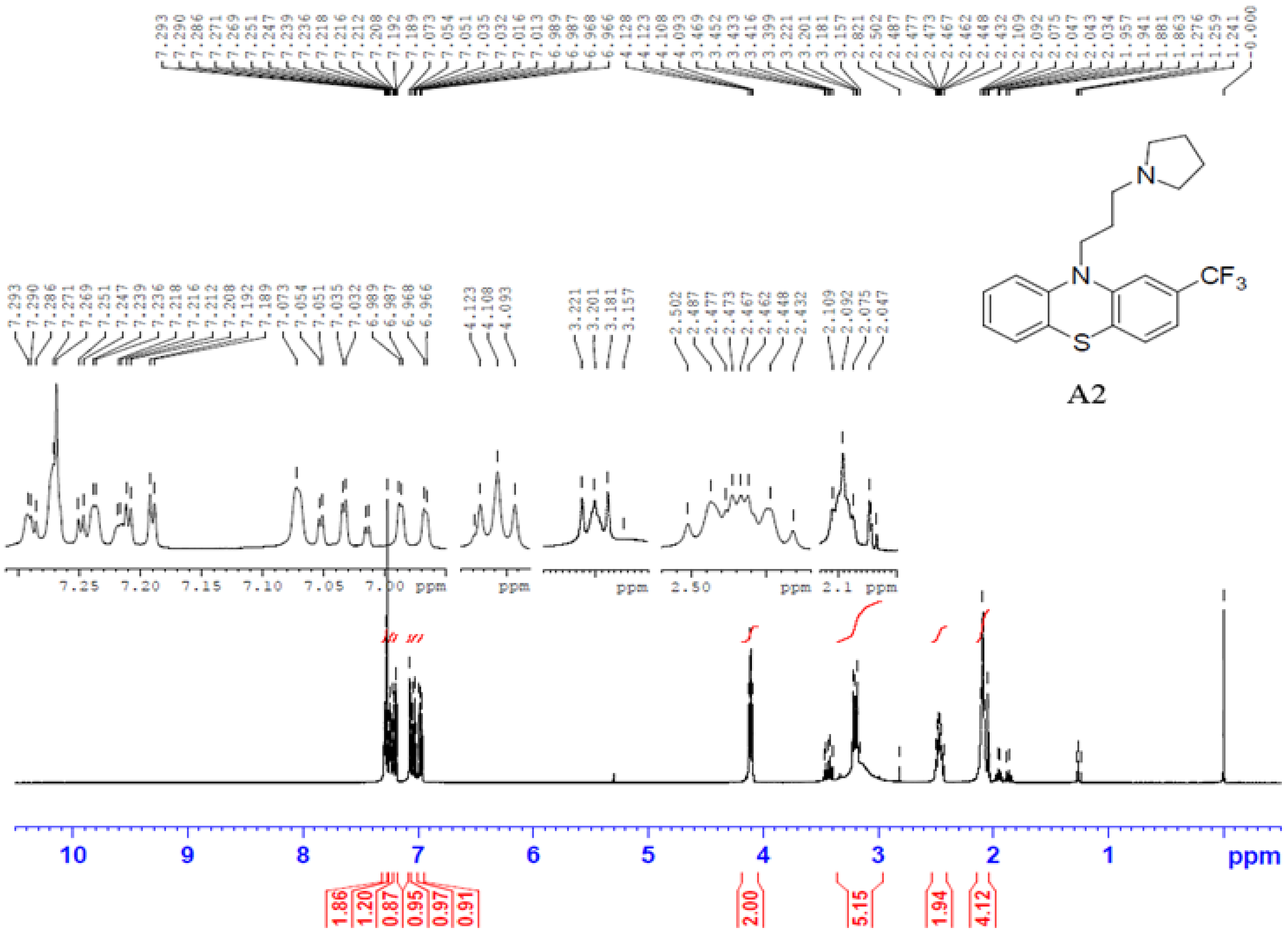

A2

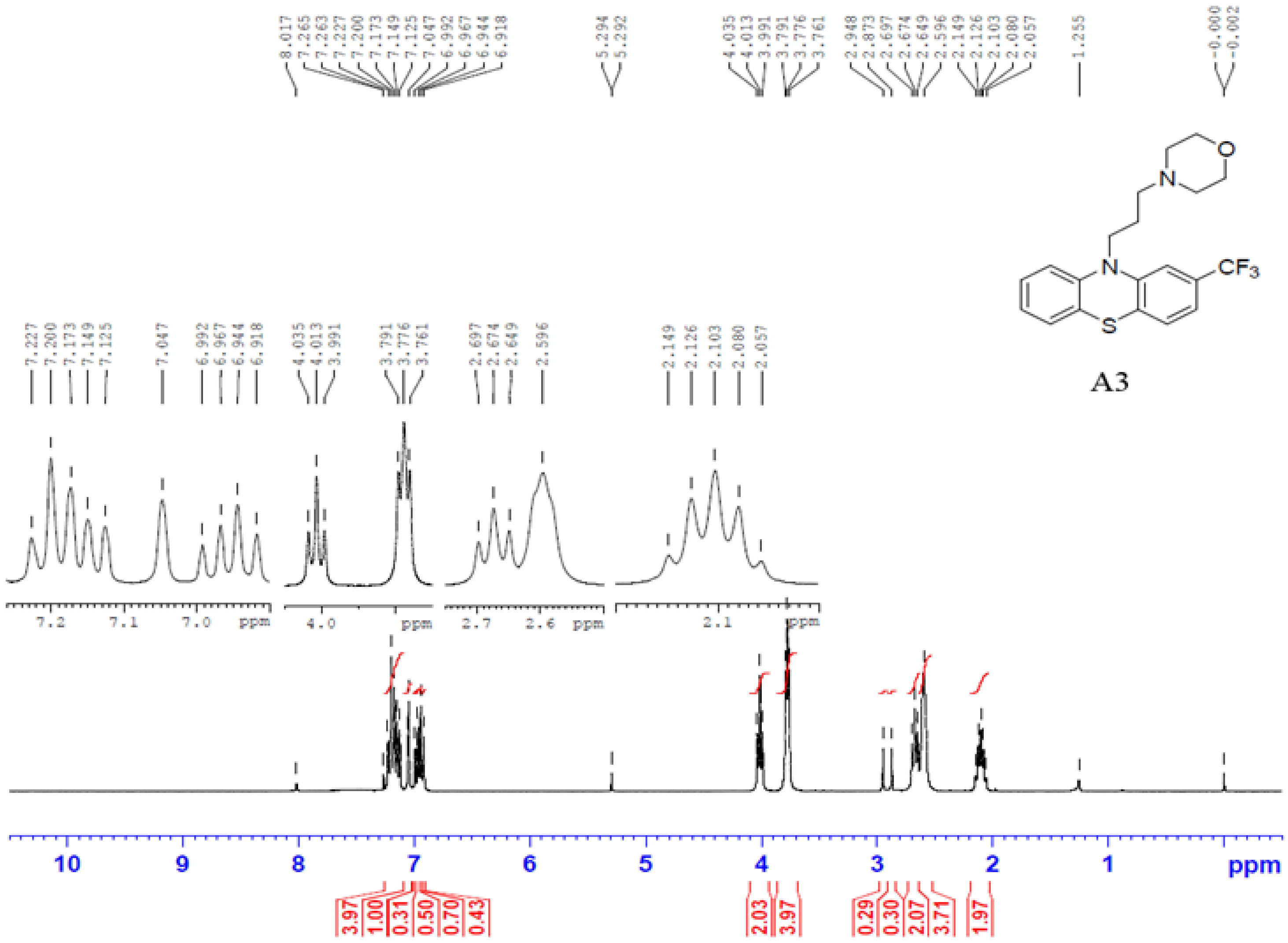
A3
CF3
N
O
S
ppm

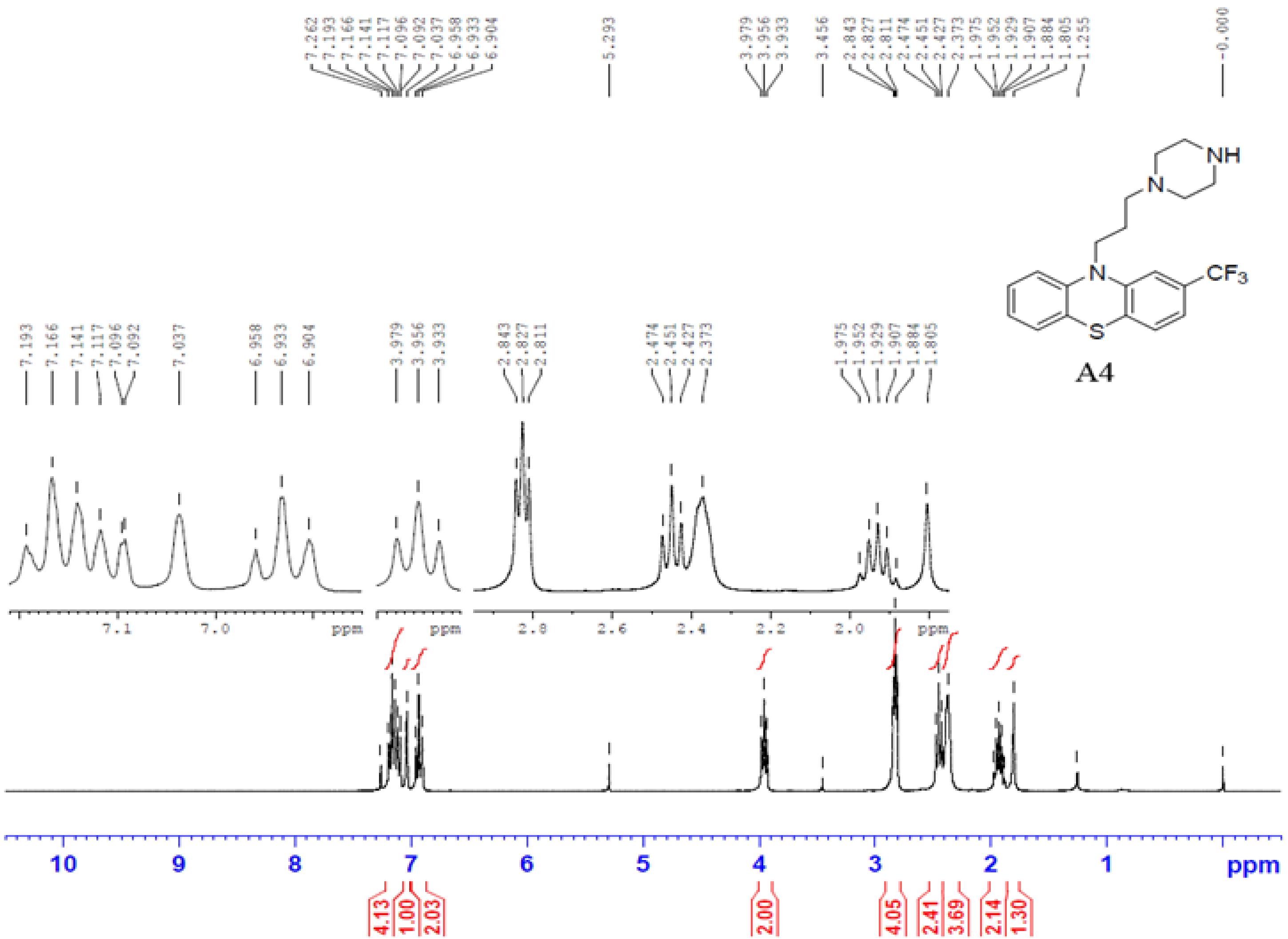

A4

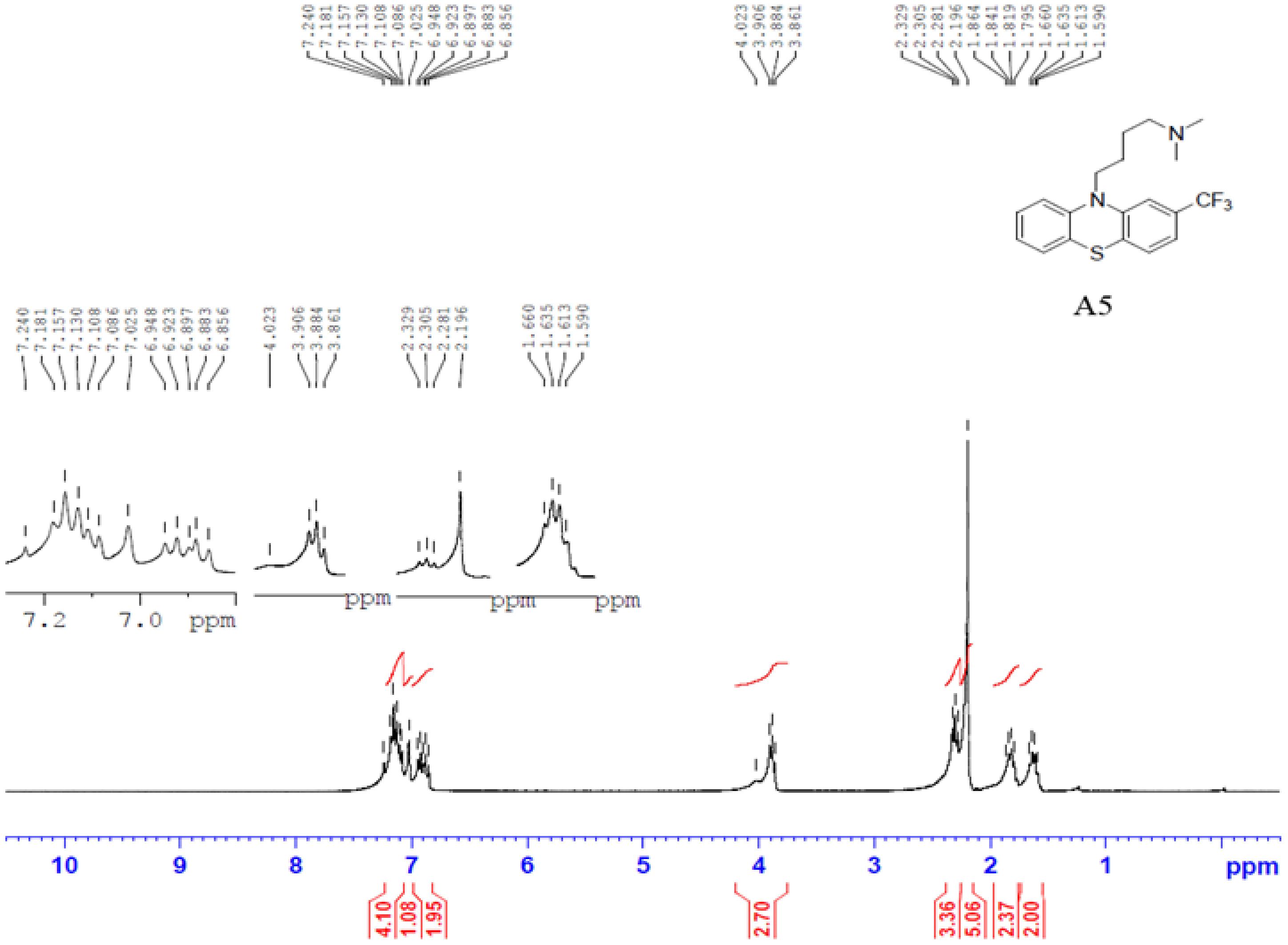
7.240
7.181
7.157
7.130
7.108
7.086
7.025
6.948
6.923
6.897
6.883
6.856
4.023
3.906
3.884
3.861
2.329
2.305
2.281
2.196
1.864
1.841
1.819
1.795
1.660
1.635
1.613
1.590
N
CF3
S
7.2
7.0
ppm
10
9
8
7
6
5
4
3
2
1
ppm
4.10
1.08
1.95
2.70
3.36
5.06
2.37
2.00

A5

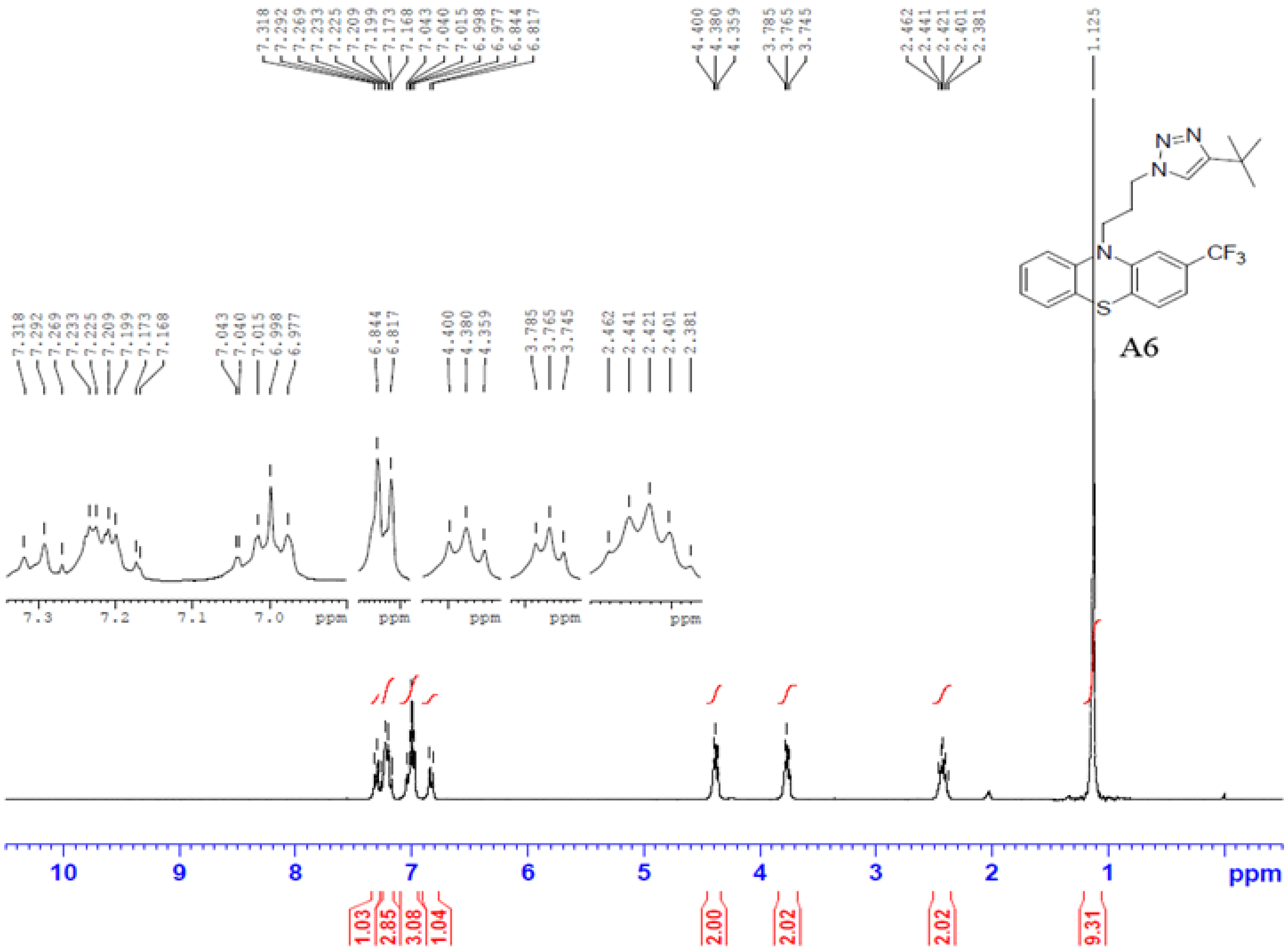

A6
ppm

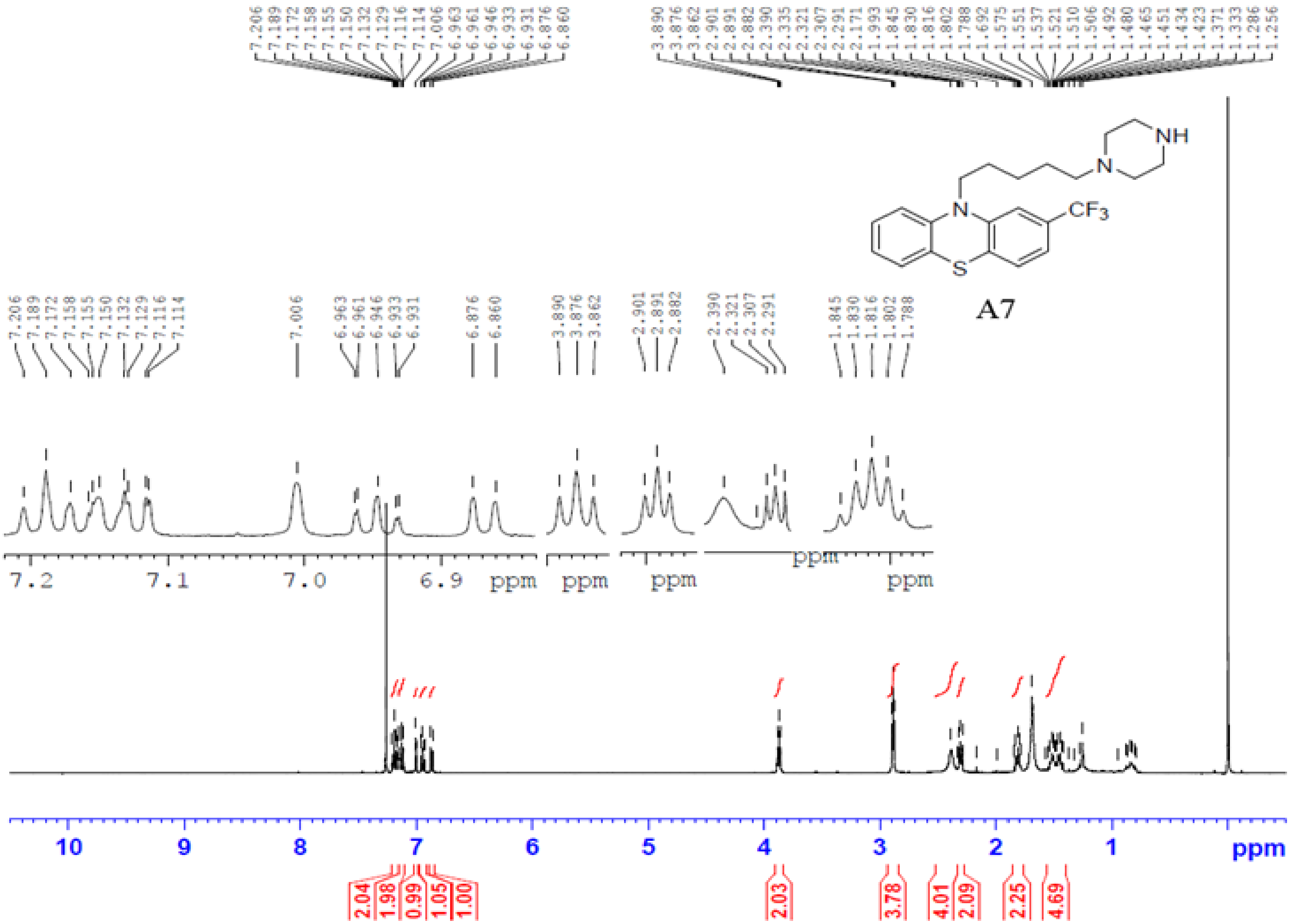
A7
ppm

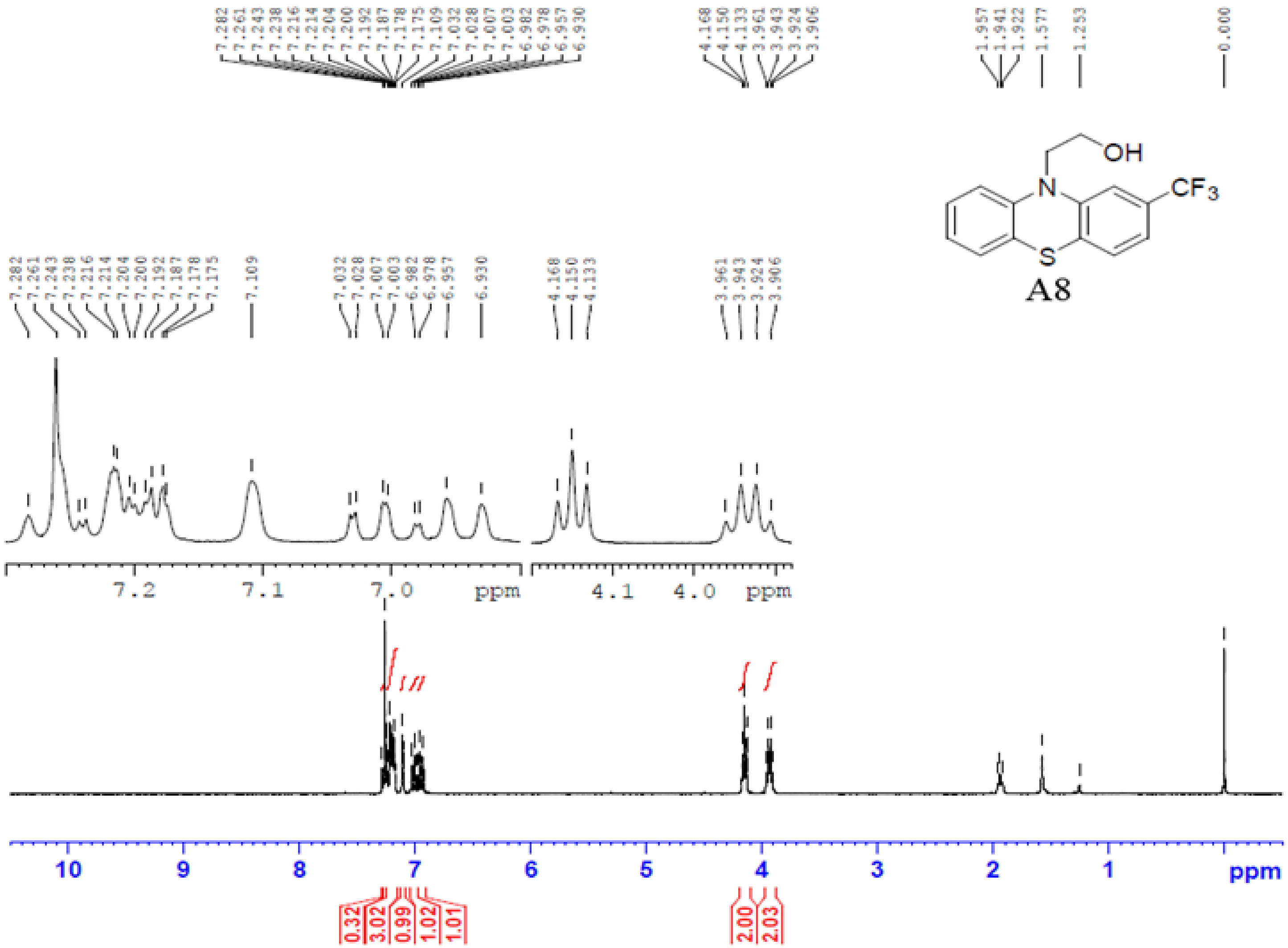
OH
N
CF3
S
A8
7.2
7.1
7.0
ppm
4.1
4.0
ppm
10
9
8
7
6
5
4
3
2
1
ppm
0.32
3.02
0.99
1.02
1.01
2.00
2.03

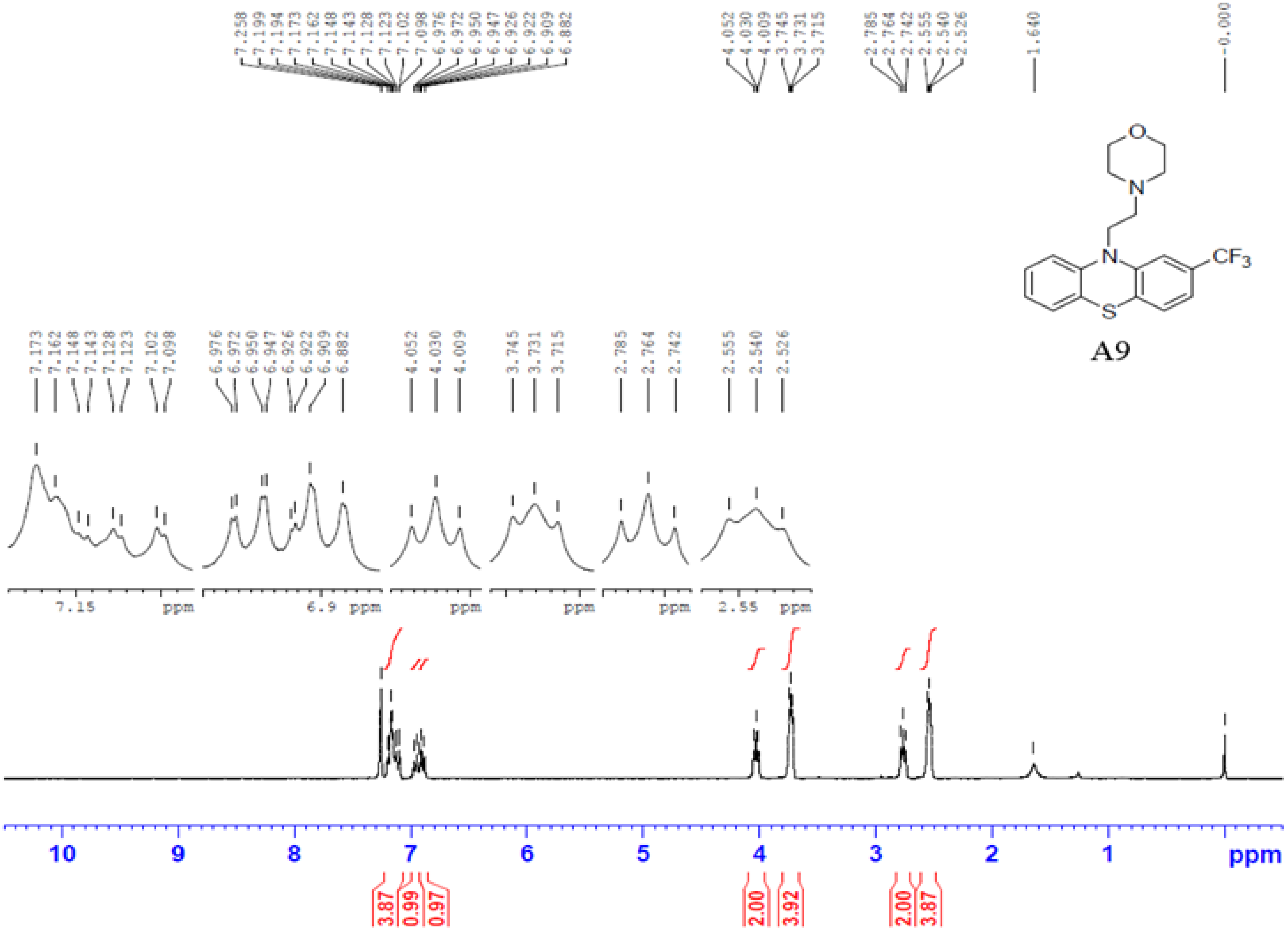

A9

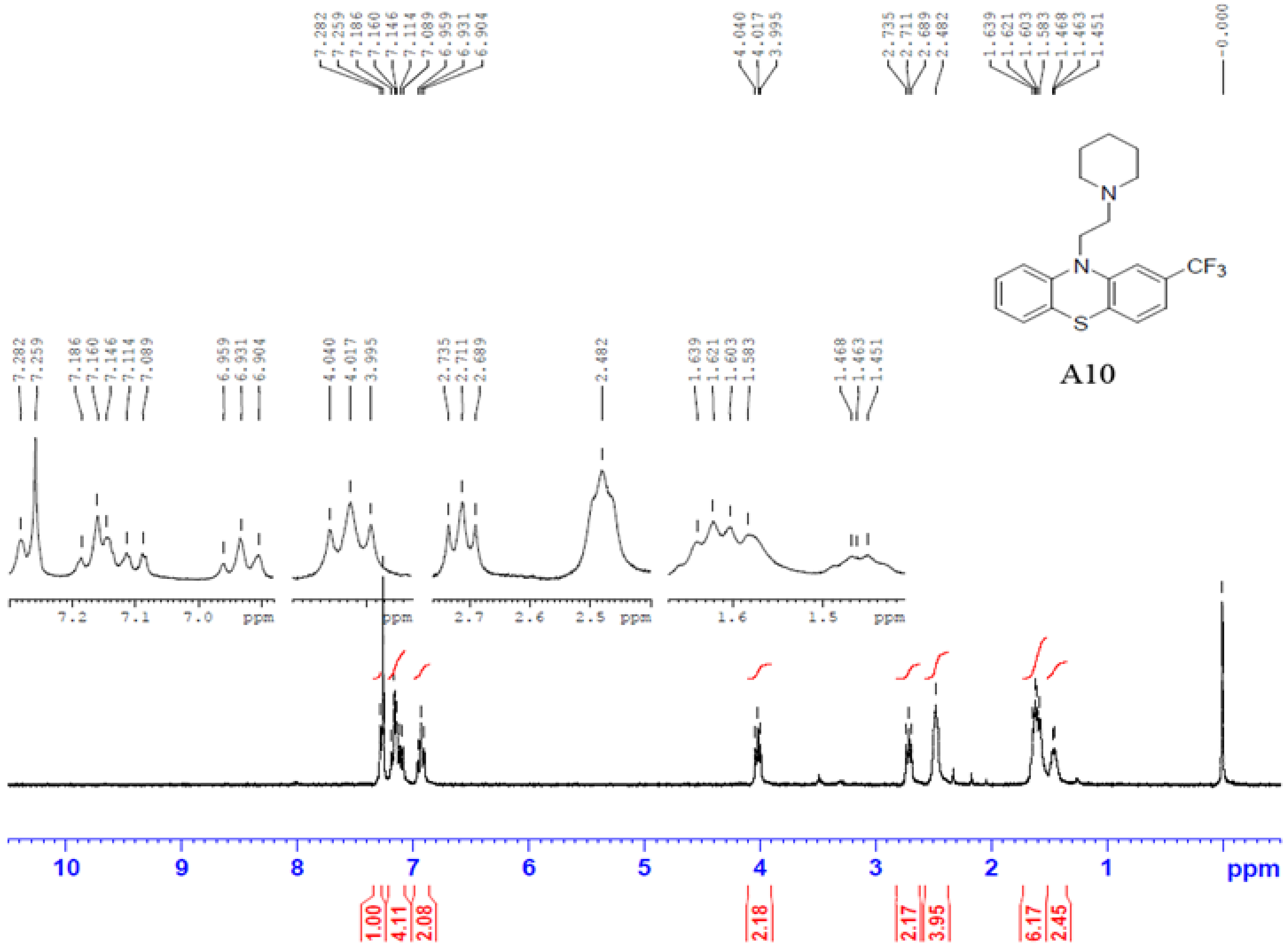
A10
CF3
N
S
ppm
10
9
8
7
6
5
4
3
2
1
1.00
4.11
2.08
2.18
2.17
3.95
6.17
2.45
7.282
7.259
7.186
7.160
7.146
7.114
7.089
6.959
6.931
6.904
4.040
4.017
3.995
2.735
2.711
2.689
2.482
1.639
1.621
1.603
1.583
1.468
1.463
1.451
0.000

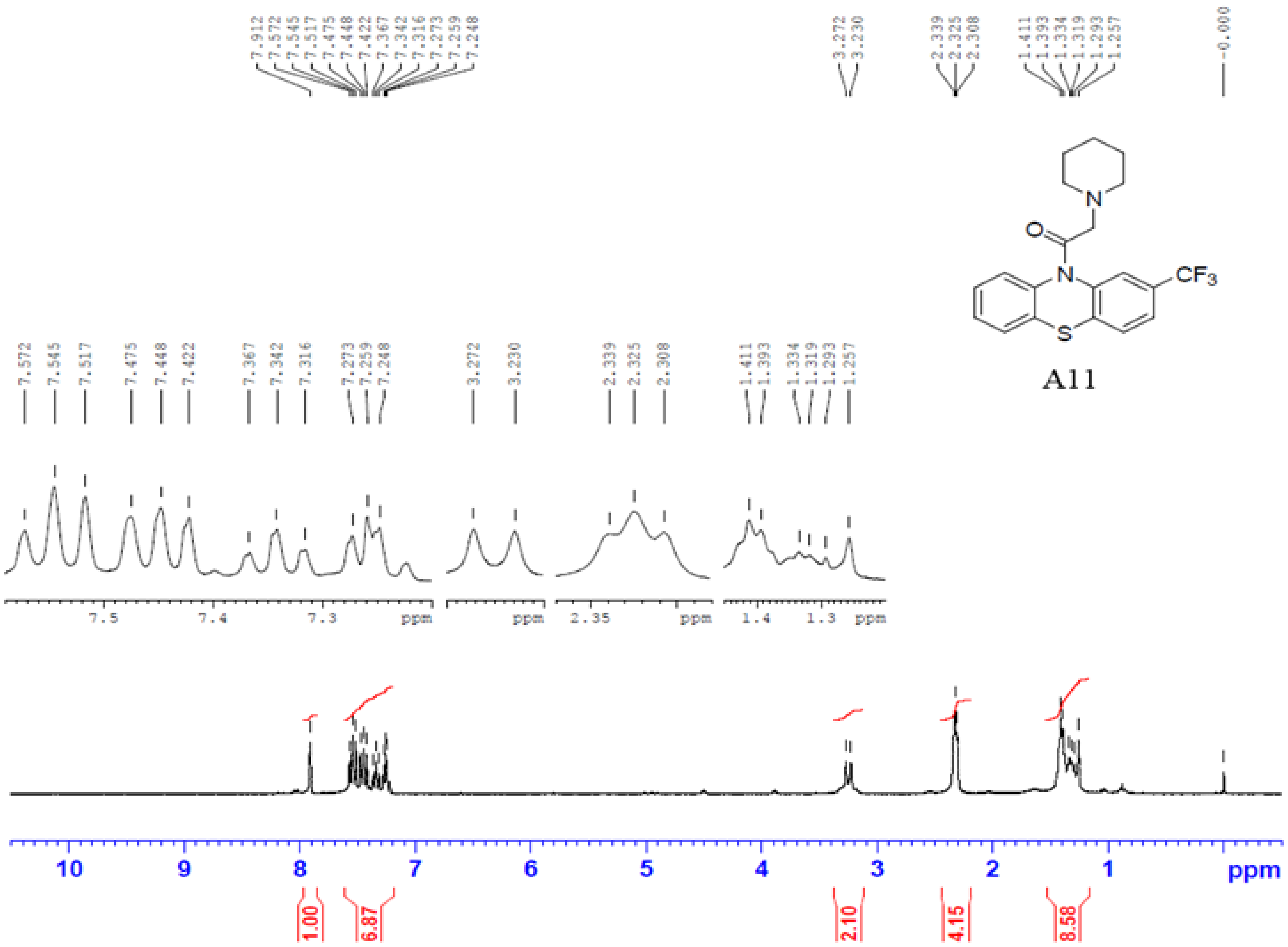

A11

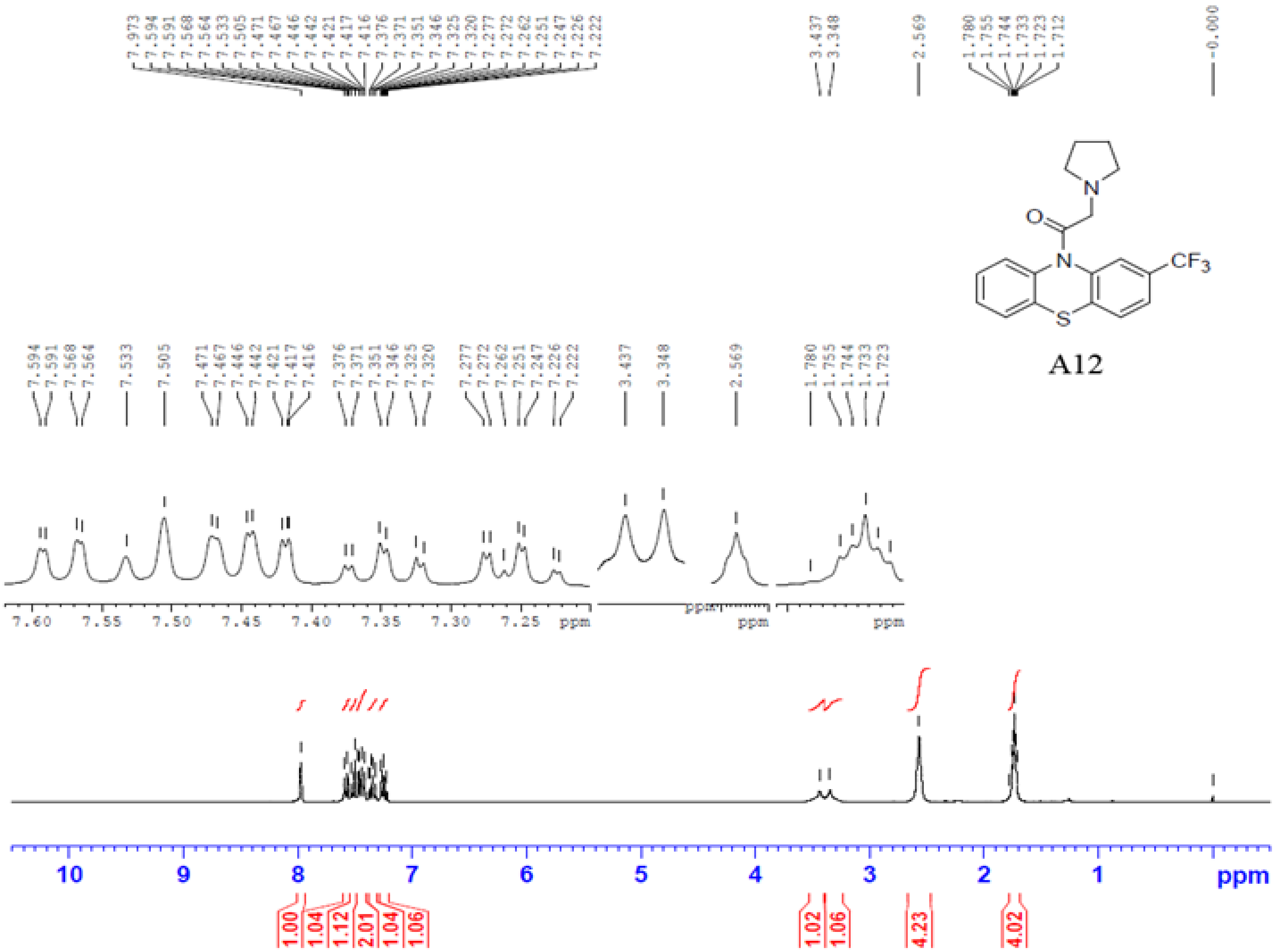
7.973 7.594 7.591 7.568 7.564 7.533 7.505 7.471 7.467 7.446 7.442 7.421 7.417 7.416 7.376 7.371 7.351 7.346 7.325 7.320 7.277 7.272 7.262 7.251 7.247 7.226 7.222
3.437 3.348
2.569
1.780 1.755 1.744 1.733 1.723 1.712
-0.000
N
O
CF3
S
A12
7.60 7.55 7.50 7.45 7.40 7.35 7.30 7.25 ppm
10 9 8 7 6 5 4 3 2 1 ppm
1.00 1.04 1.12 2.01 1.04 1.06
1.02 1.06
4.23
4.02

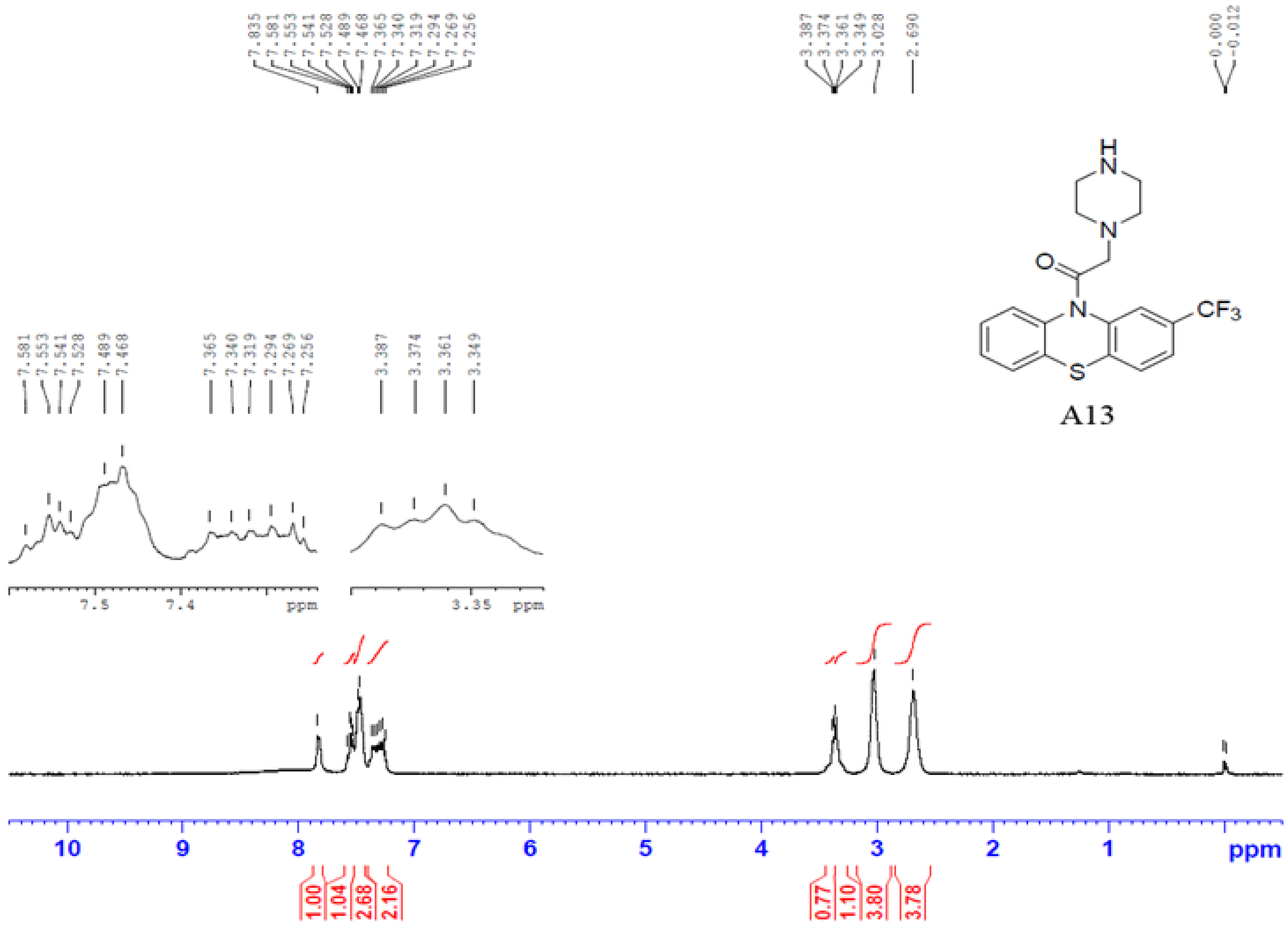
A13
CF3
ppm

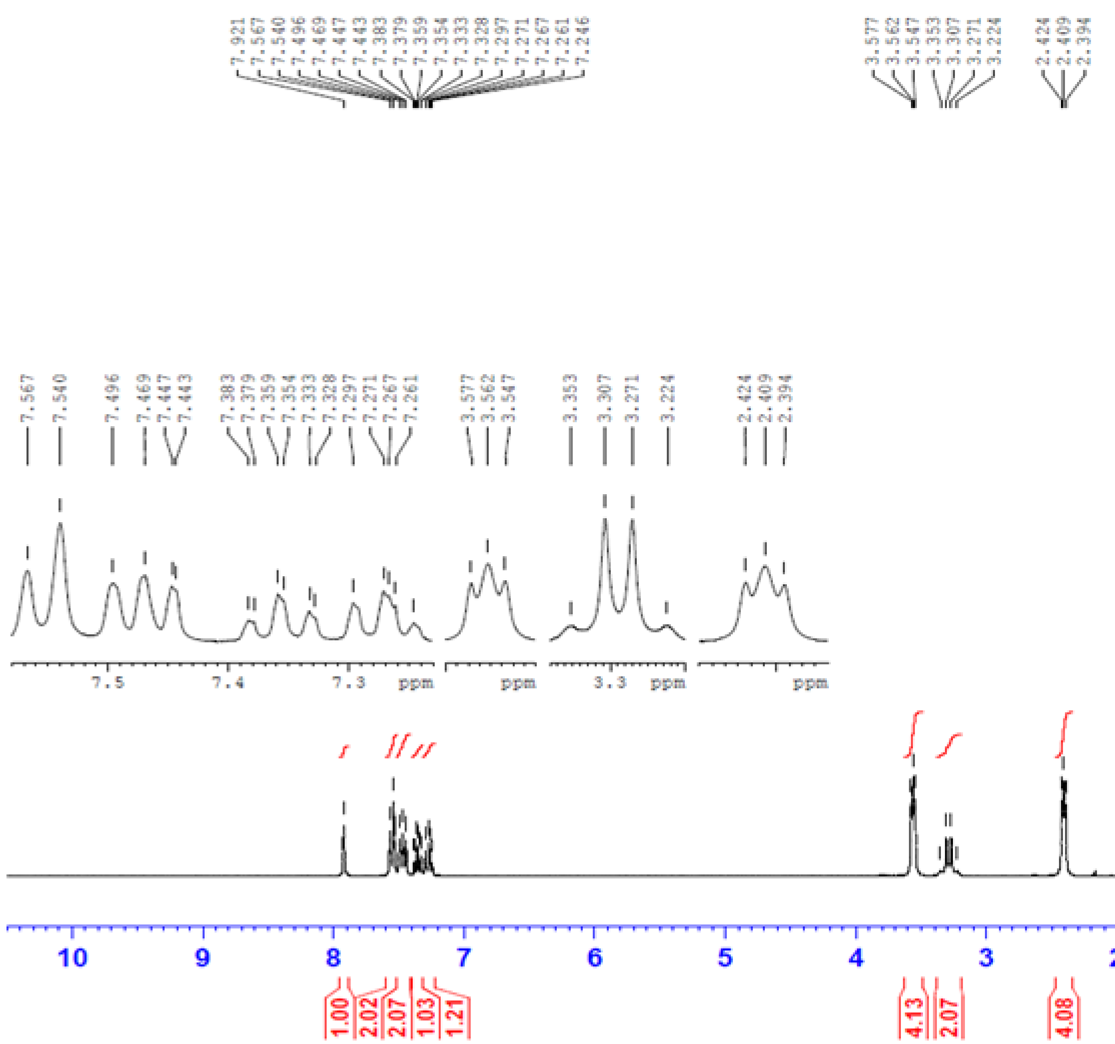

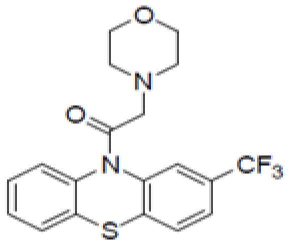

A14

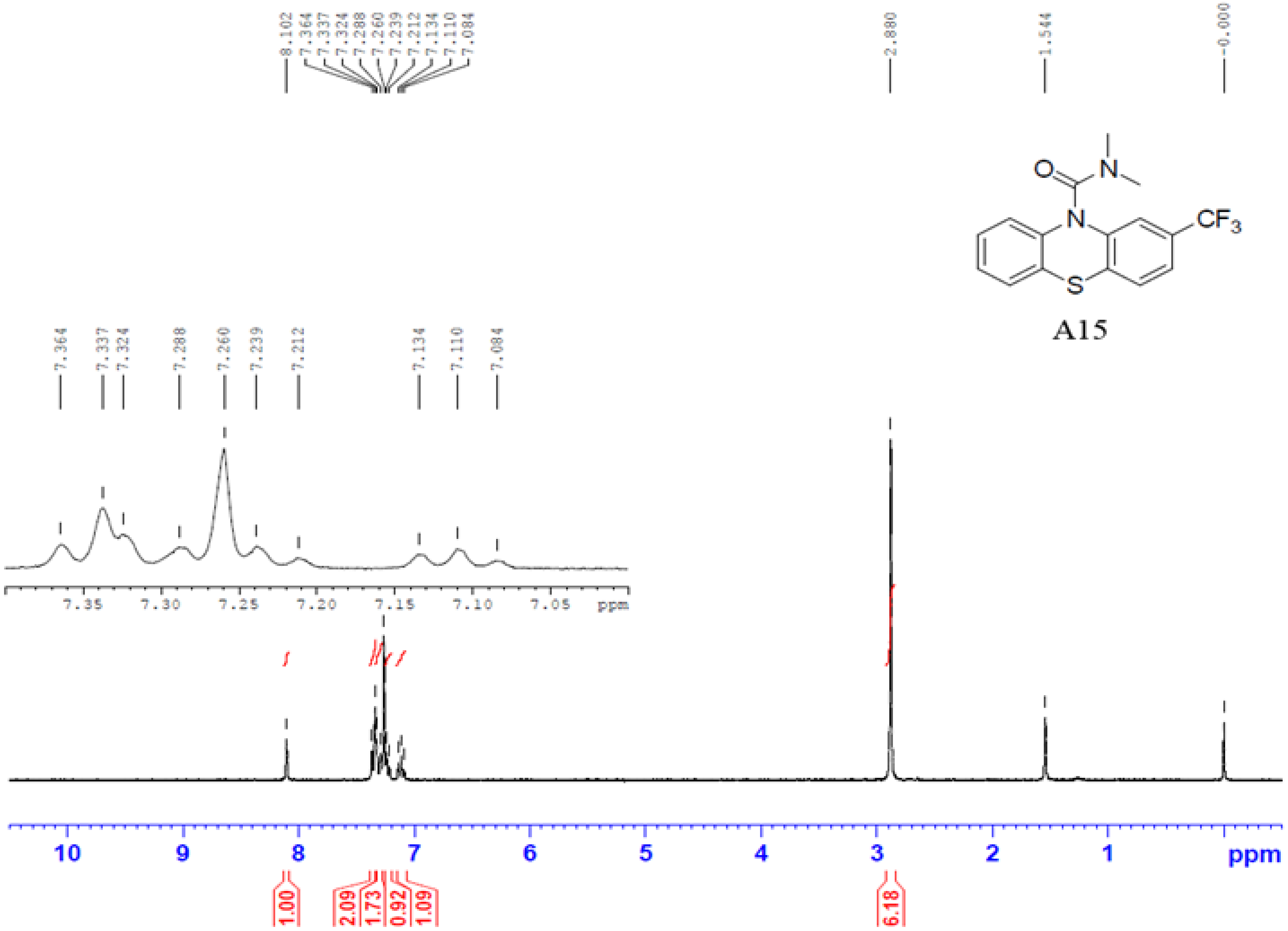

8.102
7.364
7.337
7.324
7.288
7.260
7.239
7.212
7.134
7.110
7.084
2.880
1.544
-0.000
O
N
N
CF3
S
7.35 7.30 7.25 7.20 7.15 7.10 7.05 ppm
10 9 8 7 6 5 4 3 2 1 ppm
1.00
2.09
1.73
0.92
1.09
6.18

A15

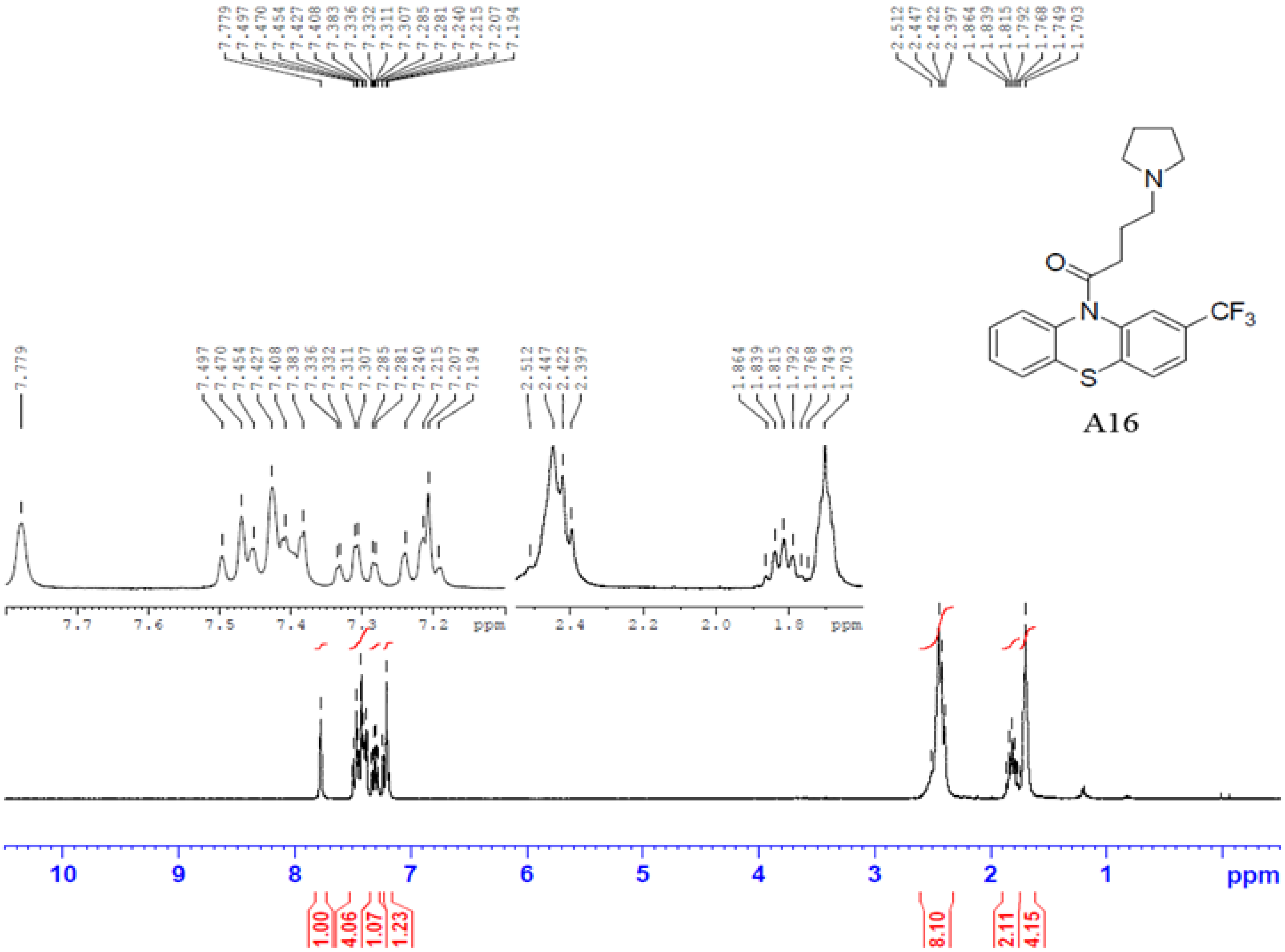

A16

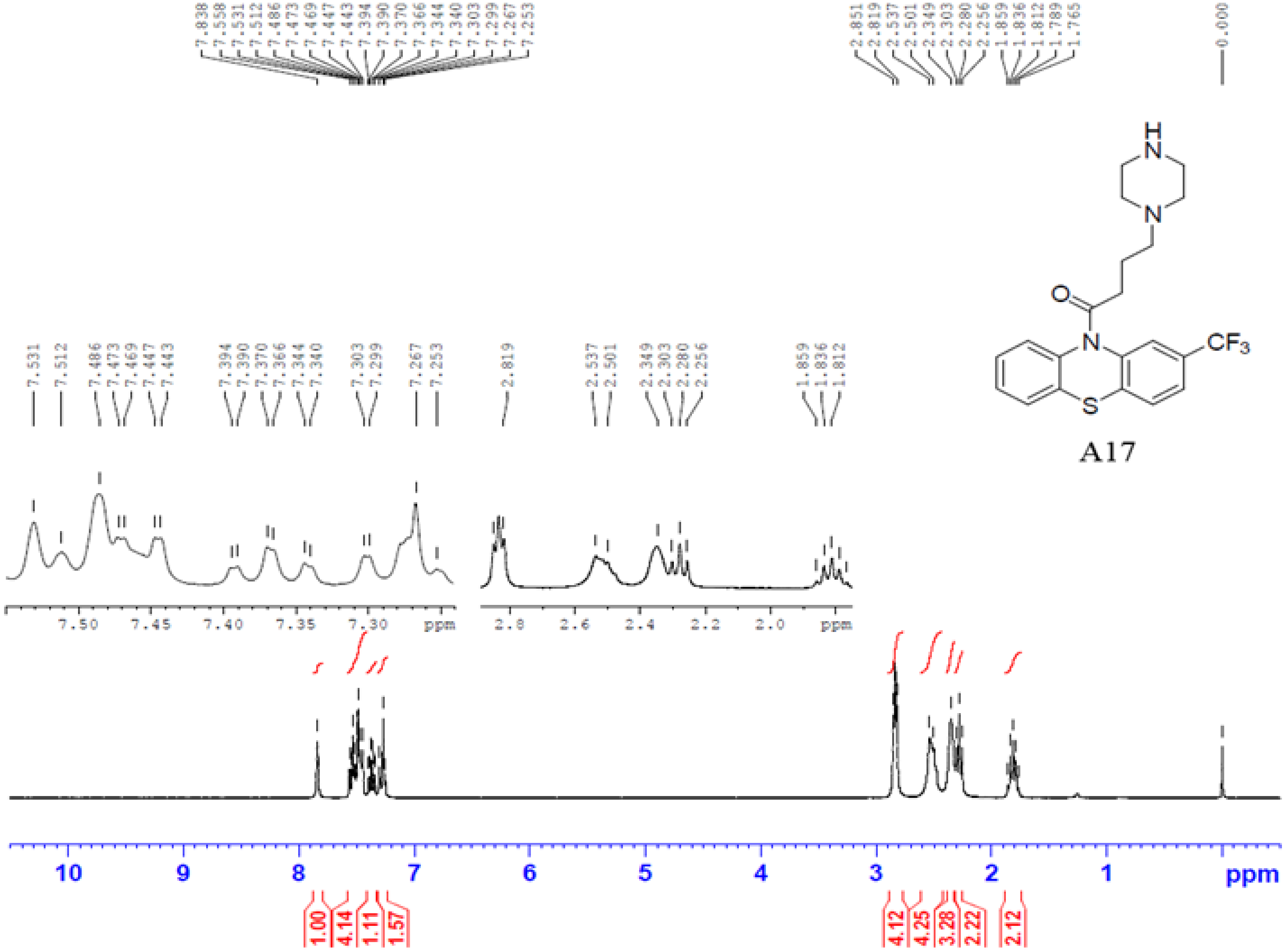

7.838 7.558 7.531 7.512 7.486 7.473 7.469 7.447 7.443 7.394 7.390 7.370 7.366 7.344 7.340 7.303 7.299 7.267 7.253
2.851 2.819 2.537 2.501 2.349 2.303 2.280 2.256 1.859 1.836 1.812 1.789 1.765
0.000
CF3
O
N
S
H
1.00 4.14 1.11 1.57
4.12 4.25 3.28 2.22 2.12
ppm

A17

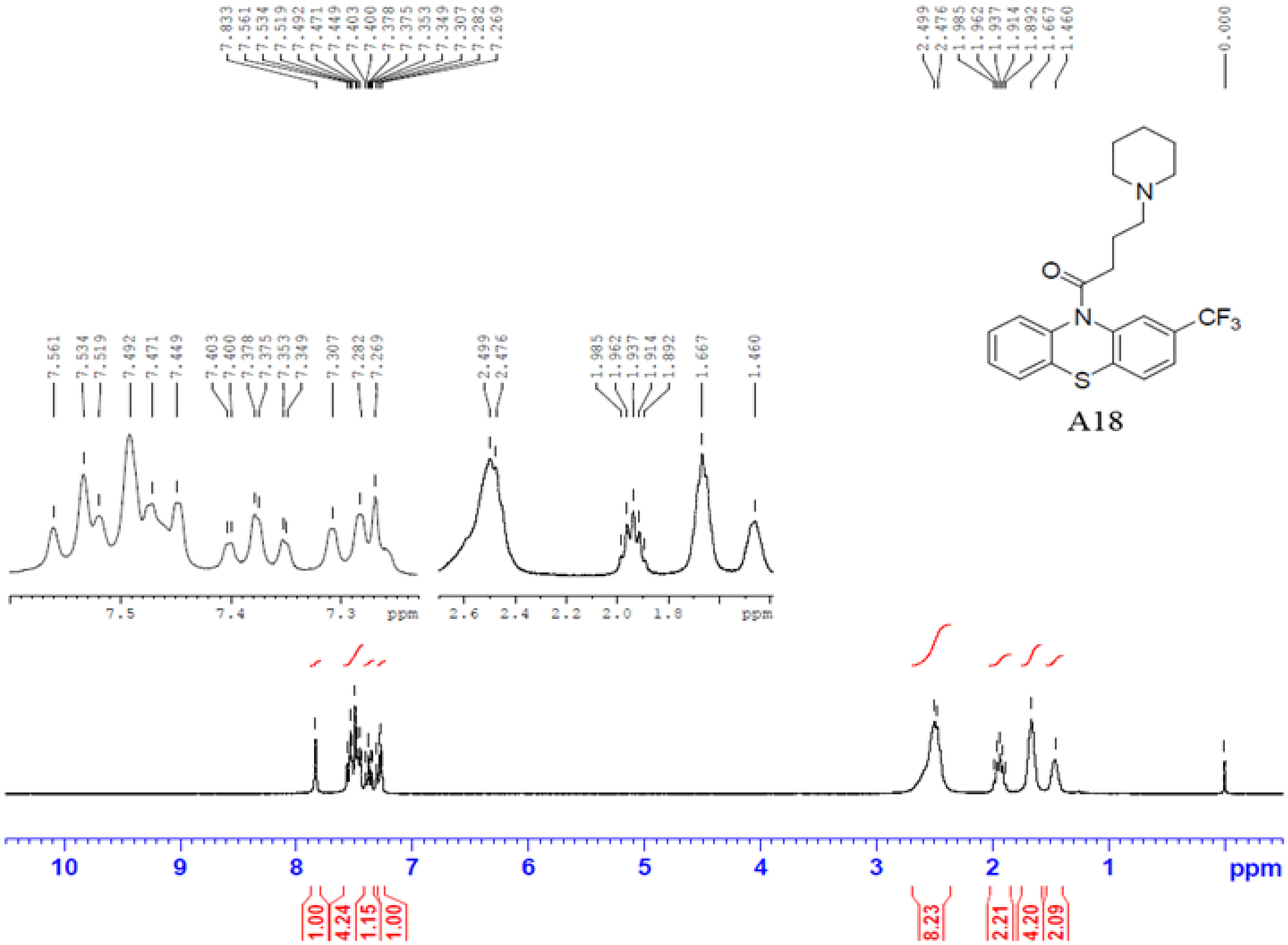
7.833
7.561
7.534
7.519
7.492
7.471
7.449
7.403
7.400
7.378
7.375
7.353
7.349
7.307
7.282
7.269
2.499
2.476
1.985
1.962
1.937
1.914
1.892
1.667
1.460
0.000
ppm
1.00
4.24
1.15
1.00
8.23
2.21
4.20
2.09
N
O
N
CF$_3$
S

A18

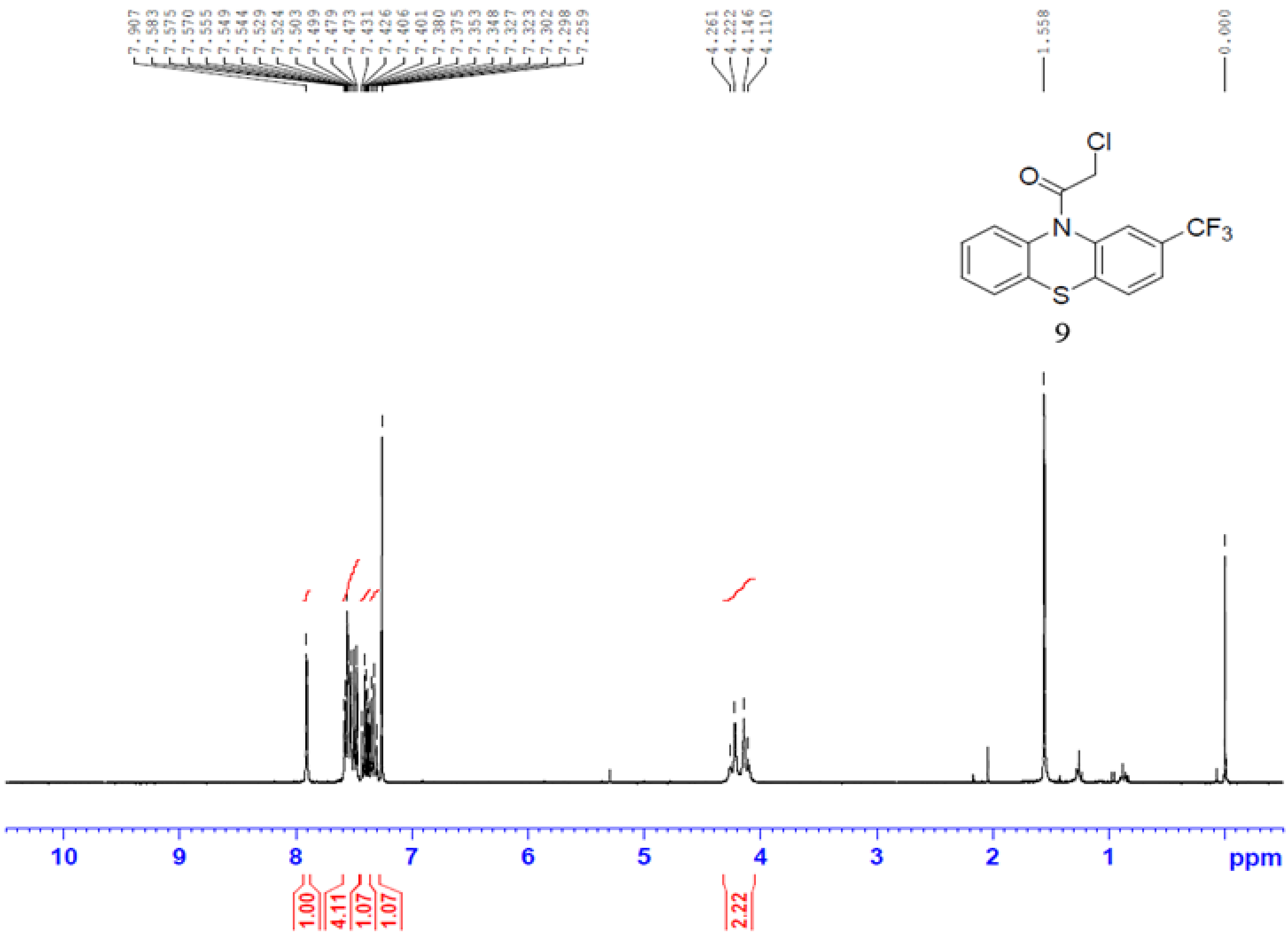
7.907
7.583
7.575
7.570
7.555
7.549
7.544
7.529
7.524
7.503
7.499
7.479
7.473
7.431
7.426
7.406
7.401
7.380
7.375
7.353
7.348
7.327
7.323
7.302
7.298
7.259
4.261
4.222
4.146
4.110
1.558
0.000
Cl
O
N
CF3
S
9
1.00
4.11
1.07
1.07
2.22
10
9
8
7
6
5
4
3
2
1
ppm

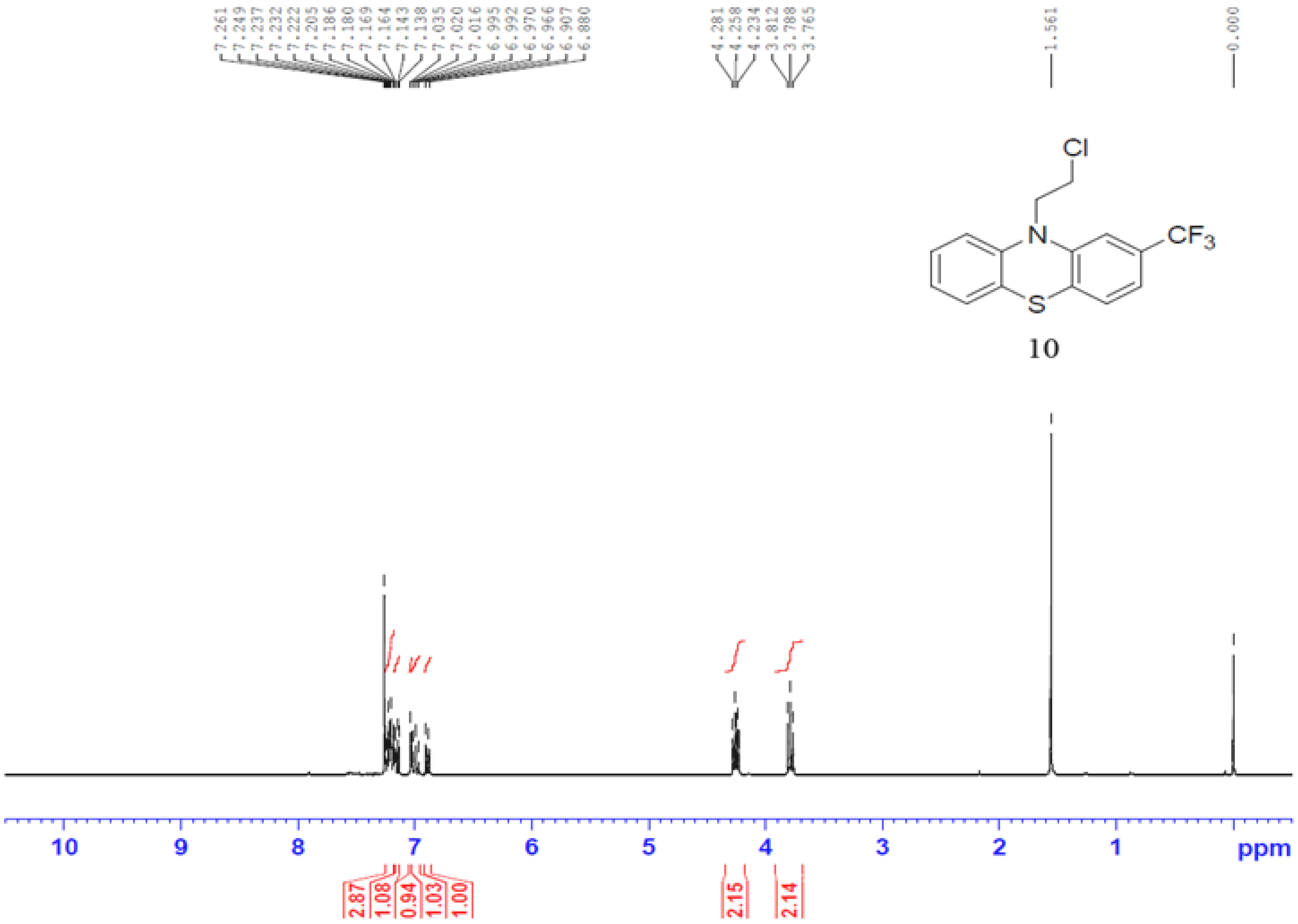

Cl
N
CF3
S
10
ppm

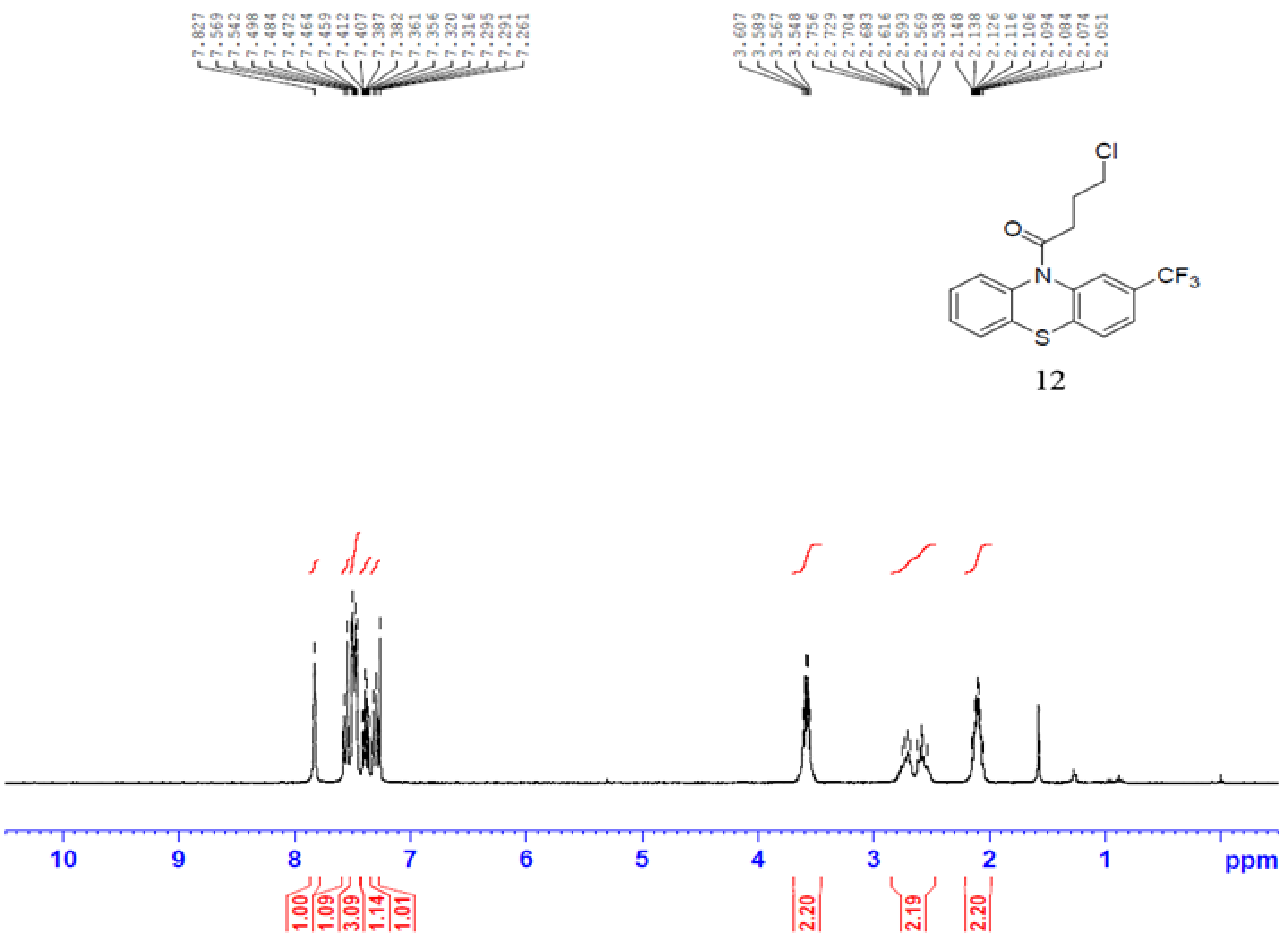
7.827 7.569 7.542 7.498 7.484 7.472 7.464 7.459 7.412 7.407 7.387 7.382 7.361 7.356 7.320 7.316 7.295 7.291 7.261
3.607 3.589 3.567 3.548 2.756 2.729 2.704 2.683 2.616 2.593 2.569 2.538 2.148 2.138 2.126 2.116 2.106 2.094 2.084 2.074 2.051
Cl
O
N
CF_3
S
12
10 9 8 7 6 5 4 3 2 1 ppm
1.00 1.09 3.09 1.14 1.01
2.20
2.19
2.20

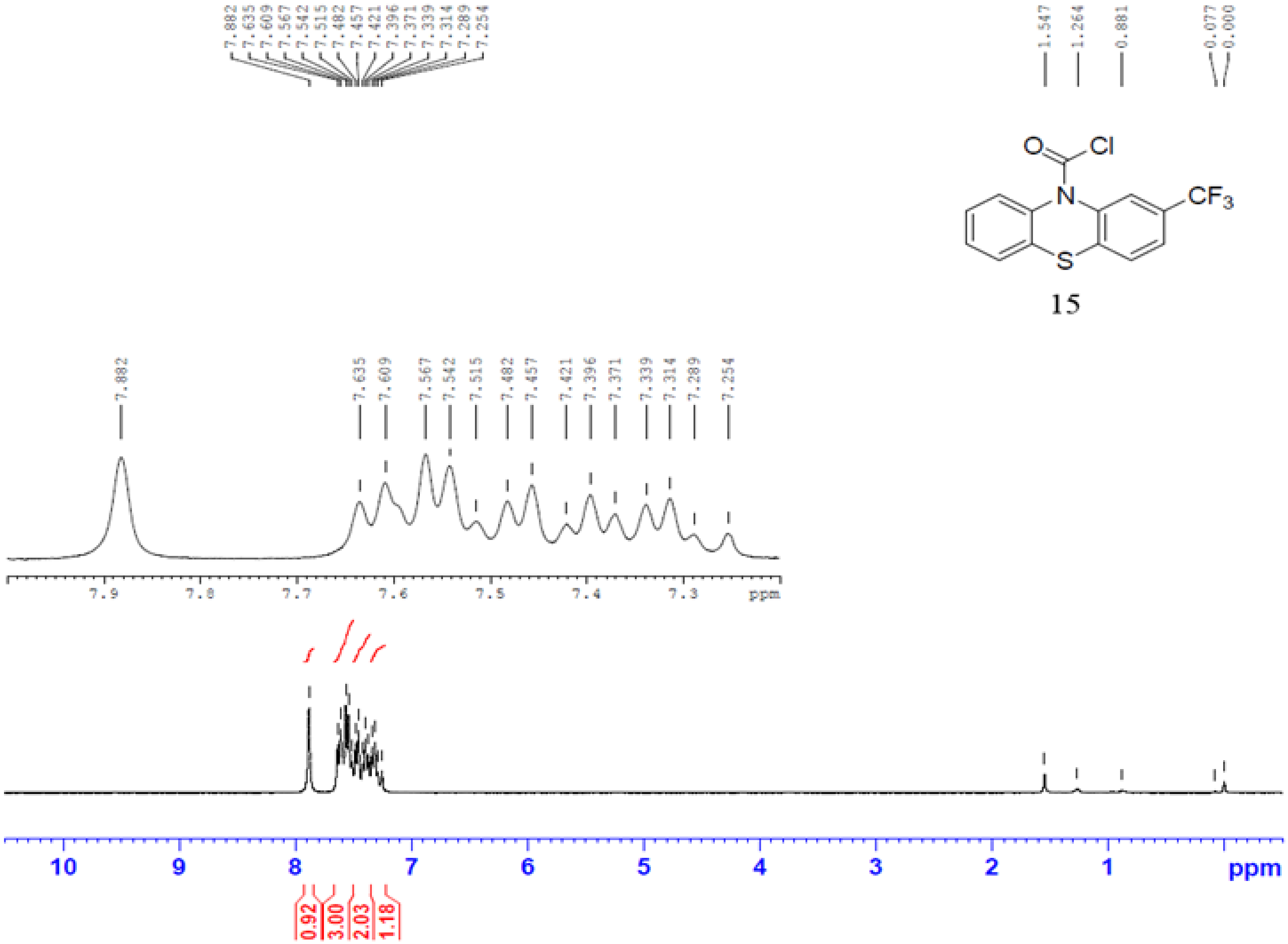
7.882
7.635
7.609
7.567
7.542
7.515
7.482
7.457
7.421
7.396
7.371
7.339
7.314
7.289
7.254
1.547
1.264
0.881
0.077
0.000
O
Cl
N
CF3
S
7.9
7.8
7.7
7.6
7.5
7.4
7.3
ppm
10
9
8
7
6
5
4
3
2
1
ppm
0.92
3.00
2.03
1.18

15
